# Association of sudden sensorineural hearing loss with meteorological factors: a time series study in Hefei, China, and a literature review

**DOI:** 10.1007/s11356-024-33943-1

**Published:** 2024-06-17

**Authors:** Xiao-Bo Li, Yan-Xun Han, Zi-Yue Fu, Yu-Chen Zhang, Min Fan, Shu-Jia Sang, Xi-Xi Chen, Bing-Yu Liang, Yu-Chen Liu, Peng-Cheng Lu, Hua-Wei Li, Hai-Feng Pan, Jian-Ming Yang

**Affiliations:** 1 Department of Otolaryngology, Head and Neck Surgery, Second Affiliated Hospital of Anhui Medical University, No. 678 Furong Road, Hefei, Anhui 230601 People’s Republic of China; 2 Department of Otolaryngology, Head and Neck Surgery, The First Affiliated Hospital of Anhui Medical University, 218 Jixi Road, Hefei, Anhui China; 3 Department of Clinical Medicine, Anhui Medical University, 81 Meishan Road, Hefei, Anhui China; 4 Institute and Otorhinolaryngology, Eye & ENT Hospital, State Key Laboratory of Medical Neurobiology, NHC Key Laboratory of Hearing Medicine Research, Fudan University, Shanghai, 200032 People’s Republic of China; 5 Department of Epidemiology and Biostatistics, School of Public Health, Anhui Medical University, 81 Meishan Road, Hefei, Anhui China

**Keywords:** Sudden sensorineural hearing loss, Climatic variables, Air pollutants, Outpatient, Mechanism, Time-series analysis

## Abstract

Air pollution can cause disease and has become a major global environmental problem. It is currently believed that air pollution may be related to the progression of SSNHL. As a rapidly developing city in recent years, Hefei has serious air pollution. In order to explore the correlation between meteorological variables and SSNHL admissions, we conducted this study. This study investigated the short-term associations between SSNHL patients admitted to the hospital and Hefei climatic variables. The daily data on SSNHL-related hospital admissions and meteorological variables containing mean temperature (T-mean; °C), diurnal temperature range (DTR; °C), atmospheric pressure (AP; Hp), and relative humidity (RH; %), from 2014 to 2021 (2558 days), were collected. A time-series analysis integrating distributed lag non-linear models and generalized linear models was used. PubMed, Embase, Cochrane Library, and Web of Science databases were searched. Literature published up to August 2023 was reviewed to explore the potential impact mechanisms of meteorological factors on SSNHL. The mechanisms were determined in detail, focusing on wind speed, air pressure, temperature, humidity, and air pollutants. Using a median of 50.00% as a baseline, the effect of exceedingly low T-mean in the single-day hysteresis effect model began at a lag of 8 days (RR = 1.032, 95% CI: 1.001 ~ 1.064). High DTR affected the admission rate for SSNHL on lag 0 day. The significance of the effect was the greatest on that day (RR = 1.054, 95% CI: 1.007 ~ 1.104) and then gradually decreased. High and exceedingly high RH affected the admission rate SSNHL on lag 0 day, and these effects lasted for 8 and 7 days, respectively. There were significant associations between all grades of AP and SSNHL. This is the first study to assess the effect of meteorological variables on SSNHL-related admissions in China using a time-series approach. Long-term exposures to high DTR, RH values, low T-mean values, and all AP grades enhance the incidence of SSNHL in residents. Limiting exposure to extremes of ambient temperature and humidity may reduce the number of SSNHL-related hospital visits in the region. It is advisable to maintain a suitable living environment temperature and avoid extreme temperature fluctuations and high humidity. During periods of high air pollution, it is recommended to stay indoors and refrain from outdoor exercise.

**Supplementary Information:**

The online version contains supplementary material available at 10.1007/s11356-024-33943-1.

## Introduction

Sudden sensorineural hearing loss (SSNHL) is defined as a hearing loss of at least 30 dB affecting three or more consecutive frequencies over 3 days for unknown reasons. It is commonly, but not always, accompanied by tinnitus and/or vertigo. Every year, five to 27 per 100,000 persons are affected by SSNHL, and approximately 66,000 new cases occur in the “”United States (Chandrasekhar et al. 2019). In China, the estimated annual incidence of SSNHL was 19 per 100,000 people (Chandrasekhar et al. 2019; Xie et al. 2020). Population studies of sudden sensorineural hearing loss have shown the occurrence of this condition in a broad age range with an average age of 50–60 years and no sex preference. The hearing loss is unilateral, with fewer than 5% of instances reporting bilateral involvement (Oh et al. 2007). The categories of hearing loss severity include mild, moderate, and severe-profound hearing loss (Nieman and Oh 2020). The hearing loss might impact high, low, or all frequencies depending on how it is configured (Moore 2016). Approximately 80% of patients have tinnitus, and about 30% experience vertigo, which is indicative of a related peripheral vestibular dysfunction (Nosrati-Zarenoe et al. 2007). The sense of having a plugged or numb ear is another typical ailment. Up to 80% of patients report a feeling of ear fullness (Sakata and Kato 2006). SSNHL may appear as a standalone issue, as a systemic disease’s presenting symptom, or during an established diagnosis. The complex etiology of SSNHL is still unknown, although risk factors for SSNHL include viral infection, environmental or occupational factors (such as loud noises, heavy metals, and organic solvents), autoimmune diseases, cardiovascular diseases, accidental events, endothelial dysfunction, metabolic diseases, and health habits (such as smoking and alcohol consumption) (Aimoni et al. 2010; Chau et al. 2010; Ciorba et al. 2010; Levy and Amedee 2010; Capaccio et al. 2012; Choi and Kim 2014; Quaranta et al. 2016; Umesawa et al. 2017; Chen et al. 2019; Jeong et al. 2019).

During the past 10 years, air pollution has emerged as a significant environmental problem, in both developing and developed nations. Population density, automobile emissions, agriculture, industrial emissions, power plants, and fossil fuel burning have strong positive relationships with air pollutant levels (Liu et al. 2016; Gu et al. 2019). In recent years, mounting research has suggested that several environmental variables may operate as potential risk factors for the development of SSNHL (Lee et al. 2019b; Zhang et al. 2021b; Tsai et al. 2021). Additionally, a preliminary report has indicated a seasonal pattern of SSNHL episodes. The results showed that the incidence of SSNHL was lowest in winter and highest in spring (Simani et al. 2022). However, no study has been conducted using advanced statistical models to systematically quantify and assess the influence of meteorological variables containing the temperature mean (T-mean), diurnal temperature range (DTR), atmospheric pressure (AP), relative humidity (RH), and wind speed on SSNHL, despite the previously clarified role of ambient temperature in SSNHL. To further explore the relationship between climatic conditions and SSNHL, it is very important to carry out such a systematic quantification and evaluation since it may increase our knowledge of how environmental variables affect SSNHL and provide ideas for enhancing public health.

In order to explore the correlation between meteorological variables and SSNHL admissions, we conducted a time-series analysis to further investigate if these connections varied among subpopulations (groups defined by sex, age, and position of hospital admission). In addition, we collated the previous relevant literature to deeply investigate the possible effects and mechanisms of meteorological factors on SSNHL from several aspects: wind speed, air pressure, temperature, humidity, and air pollutants. This is the first study to comprehensively measure and assess the effect of meteorological variables such as MT, DTR, AP, and RH on SSNHL-related admissions in China using a time-series approach.

## Materials and methods

### Basic information and overview of the study site

**Fig. 1:**
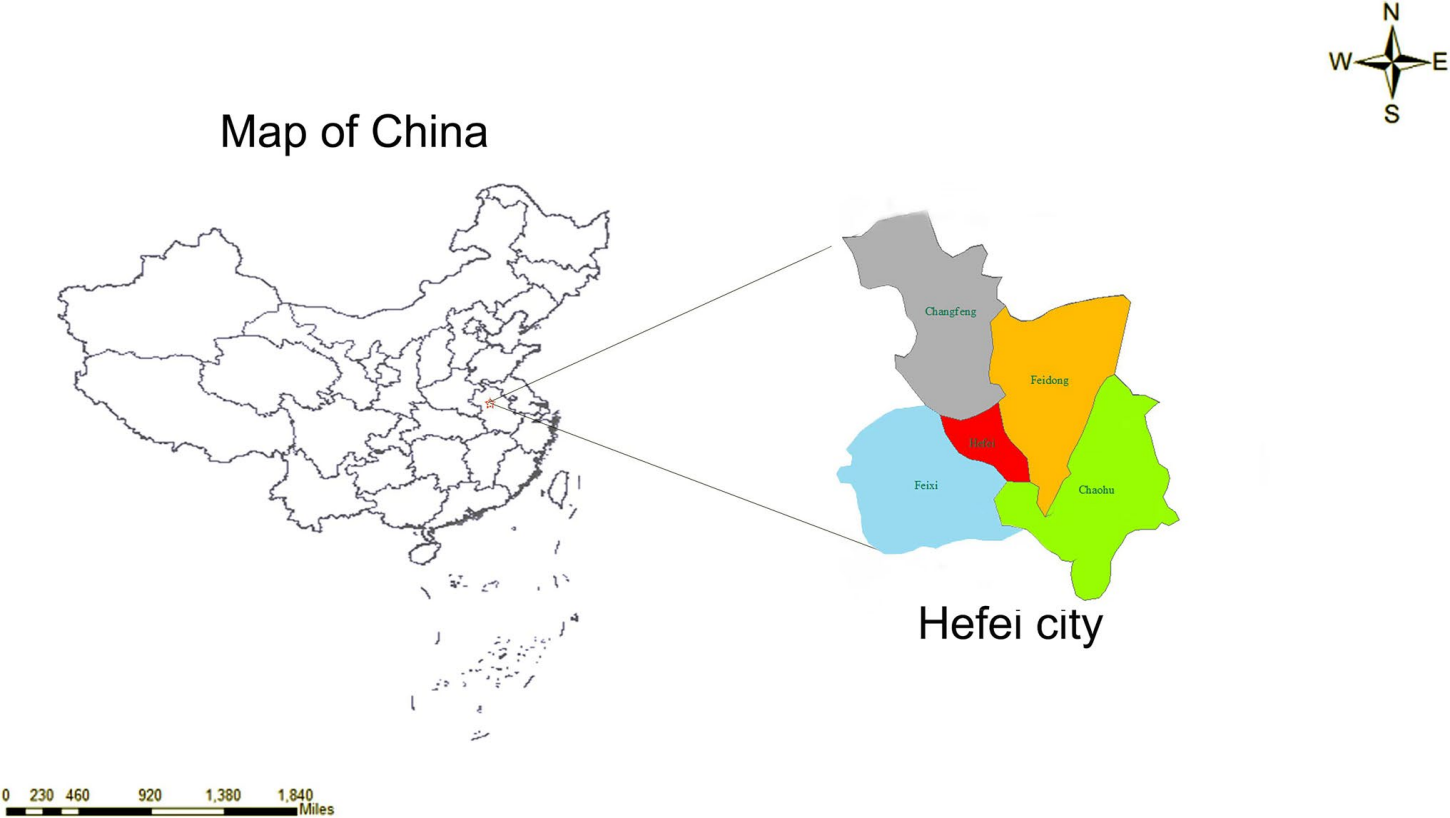
The geographical location of Hefei city

Hefei, the capital city of Anhui Province, is situated in East China within central Anhui Province (31°52′N, 117°17′E). Hefei experiences a modest annual rainfall of approximately 1000 mm, with prevailing southeasterly winds in spring and summer and northwesterly winds in autumn and winter. The city maintains an annual average temperature of 15.7 °C, with lower temperatures in winter and higher temperatures and humidity in summer due to a thicker inversion layer and slower air flow. These conditions reflect the typical subtropical monsoon climate of eastern China (Chen et al. 2023) (Fig. 1). According to the 2021 census data, Hefei has a population of 9,369,881, a built-up area of 528.5 km^2^, an urbanization rate (urban population/total population) of 82.28%, and a gross domestic product of 100.4572 billion renminbi (RMB) (China APBoSo. 21 May 2021 edn: https://zwgk.hefei.gov.cn/public/14891/106487817.html, 2019). By selecting Hefei as the focus of this study, the meteorological characteristics of cities in eastern China are accurately represented, allowing for a universal and representative correlation between meteorological variables and SSNHL.

Besides, in recent years, China has experienced rapid economic growth and accelerated urbanization, leading to a significant increase in man-made pollution issues. The Yangtze River Delta region, being the most economically developed coastal special economic zone in China, faces dense cities and highly concentrated air pollution emissions, showcasing distinct regional air pollution characteristics. This area is among the most air-polluted and densely populated regions in China (Feng et al. 2006). Hefei, as a representative city in the Yangtze River Delta region, is situated amidst the heavily polluted areas of Beijing-Tianjin-Hebei, Fenwei Plain, and the Yangtze River Delta to the north, west, and east respectively, resulting in severe cross-regional pollution. The primary pollutants contributing to air pollution in Hefei are fine particulate matter (PM2.5) and inhalable particulate matter (PM10), which could potentially impact sudden sensorineural hearing loss (SSNHL). This study not only investigates the correlation between meteorological factors and SSNHL but also delves into the connection between air pollutants and SSNHL in Hefei. Such research can offer a deeper insight into the link between air pollution and disease progression in the Yangtze River Delta region and beyond, serving as a valuable resource for clinical and air quality management practices.

### Source and collection of data

The Department of Head and Neck Surgery at the First Affiliated Hospital of Anhui Medical University and the Second Affiliated Hospital of Anhui Medical University, two of the city’s largest public head and neck surgery clinics preferred by local patients with head and neck surgery diseases, provided daily data on hospital admissions (including first admissions and readmissions) for SSNHL in Hefei from 2014 to 2021 (2,558 days). Demographic factors such as age, sex, home address, date of hospital admission, and status of hospital admission were used in the data collection for SSNHL (i.e., first admission or readmission). Hospital admissions records for patients whose demographic details (including sex and age) were not identified or whose residence locations were not in Hefei City were excluded to render the obtained data legitimate and trustworthy. The Ethical Committee of the Anhui Medical University authorized the current research, which was carried out following the Ethical Principles for Medical Research from the Declaration of Helsinki (20,131,220).

The meteorological data were collected from the Hefei Meteorological Bureau and the air pollutant data from Hefei Environmental Monitoring Center. Air pollutant data from Hefei Environmental Monitoring Center contained the daily 8-h highest ozone concentration (O_3_), particulate matter (PM_2.5_), carbonic oxide (CO), inhalable particles (PM_10_), nitrogen dioxide (NO_2_), and sulfur dioxide (SO_2_). Meteorological data from Hefei Meteorological Bureau contained RH, daily average temperature, AP, wind speed, daily maximum temperature, and daily minimum temperature, where daily maximum and minimum temperatures were used to calculate the daily average temperature difference.

### Statistical analysis

Categorical variables were presented as numbers (%). Continuous variables with a normal distribution were expressed as means (standard deviations, SD) and otherwise as medians (interquartile ranges, IQR). In addition, we removed the strongly correlated variables using the Spearman correlation test, and two variables were considered to be strongly correlated when the test result was greater than 0.7 (Shao et al. 2021). Given that environmental exposures are complex and variable, their effects on human health are often non-linear. And the flexibility of generalized additive models is widely used due to their ability to deal well with non-linear points in the environment (Ma et al. 2014; Li et al. 2022), given that the daily number of hospitalizations for SSNHL in this study is a small probability event and follows a quasi-Poisson distribution. Consistent with previous studies (Gasparrini 2014), we used a quasi-Poisson generalized additive model combined with distributed nonlinear lag model (DLNM) to explore the relationship between the admission rate for SSNHL and the climate. The time series model of meteorological factors (MF) was as follows:

$${Y}_{t}\sim Poisson({\mu }_{t})$$

$$Log\left({\mu }_{t}\right)= \alpha +\beta {MF}_{t,l}+ns({Pollutant}_{t},df)+ns(Time,df)+factor(Holiday)+factor(DOW)$$

where ${\mu }_{t}$ represents the number of daily hospitalizations for SSNHL; $\alpha$ means the intercept distance; ${MF}_{t,l}$ is the 0 ~ *l* day lag matrix of MF, *β* corresponds to the vector coefficients of MF matrix; *ns* denotes natural cubic spline function; *Pollutant* represents O_3_, PM_10_, SO_2_, NO_2_, and CO; a natural cubic spline with 8 degrees of freedom per year was used to adjust for seasonality and long-term trend. The effect of weekends and holidays was controlled for by including “DOW” and “Holiday” in the models, respectively.

The evaluation of the degree of fit and freedom of these two models was done with the Akaike information criterion and residual analysis. Based on the incubation period for SSNHL, we reviewed similar literature and determined the lag days as 2 weeks (14 days). We used RR and its 95% CI to depict cumulative and single-day risk results for the admission rate for SSNHL. Finally, the 50th percentile of MF was used as a reference to classify MF into four categories: exceedingly high (95th), high (75th), low (25th), and exceedingly low (5th).

### Software usage

The map of Hefei City, Anhui Province, China, was obtained from the software of ArcMap Desktop (10.7.0.10450 version). Descriptive analyses were performed using SPSS 23.0 and the remaining statistical analyses using R software (version 4.1.2). The matching of meteorological models in time series was implemented with “spline” and “DLNM” packages. The criteria for determining bilateral statistical differences were based on a “”*p* value < 0.05.

### Literature review

Two researchers independently searched the Cochrane Database, EMBASE, PubMed, and Web of Science. It was searched using a combination of Medical Subject Heading terms (MeSH), free-text search terms, and Boolean operators. They collected the relevant literature focused on the potential impact mechanisms of meteorological factors on SSNHL until August 2023. The search terms included sudden sensorineural hearing loss, wind speed, air pressure, temperature, humidity, and air pollutants.

The inclusion criteria are research studies that delineated the potential impact mechanisms of meteorological factors on SSNHL. The exclusion criteria are (1) reports of individual cases, evaluations, remarks, and summaries presented at conferences, commentaries, and letters without unique data and (2) data that could not be entirely extracted.

## Results

### Descriptive summary

The distribution of the daily number of patients with SSNHL, major pollutants, and MFs in Hefei, China, 2014–2021, is shown in Table 1. We identified a total of 4984 patients with SSNHL over a period of 2558 days, with an average of 1.43 patients with SSNHL per day. Among all patients with SSNHL, there were 1871 instances (51.02%) in men, 1796 (48.98%) in women, 3158 (86.15%) in the 0–65 year age group, and 508 (13.85%) in the ≥ 65 years age group. Among them, the ratio of male to female was about 1:1, and the ratio of young individuals (aged 0–65 years) to older (aged ≥ 65 years) was about 7:1. The T-mean, DTR, RH, AP, and wind speed were 17.40 °C (range: − 5.90 to 35.60 °C), 8.70 (range: 0.60–22.10 °C), 78.00% (range: 33.00–100%), 1012.10 hPA (range: 987.50–1041.00 hPA), and 1.90 m/s (range: 0.30–6.60 m/s), respectively. The average concentrations of the air pollutants PM_2.5_, PM_10_, SO_2_, NO_2_, CO, and O_3_ were 44.00 μg/m^3^ (PM_2.5_, range: 5.00–351.00 μg/m^3^), 74.00 μg/m^3^ (PM_10_, range: 11.00–413.00 μg/m^3^), 10.00 μg/m^3^ (SO_2_, range: 2.00–58.00 μg/m^3^), 36.00 μg/m^3^ (NO_2_, range: 9.00–134.00 μg/m^3^), 800.00 μg/m^3^ (CO, range: 320.00–2860.00 μg/m^3^), and 52.00 μg/m^3^ (O_3_, range: 4.00–185.00 μg/m^3^), respectively. Supplementary Fig. 1 presented the temporal trends of meteorological variables and SSNHL outpatient visits in Hefei City during the study period.

**Table 1:** Summary statistics of daily numbers of sudden deafness, meteorological conditions, and daily air pollutants in Hefei from 2014 to 2021 (2558 days)

Variables	Counts (%)	Mean ± SD	Centiles						
			Minimum	*P*_5_	*P*_25_	Median	*P*_75_	*P*_95_	Maximum
**Sudden deafness**									
Total	3667(100.00)	1.43 ± 1.85	0	0	0	1	2	5	18
Male	1871(51.02)	0.73 ± 1.14	0	0	0	0	1	3	11
Female	1796(48.98)	0.70 ± 1.08	0	0	0	0	1	3	10
0–65 years	508(13.85)	0.20 ± 0.50	0	0	0	0	0	1	5
≥ 65 years	3158(86.15)	1.23 ± 1.62	0	0	0	1	2	4	4
**Meteorological conditions**									
T-mean (℃)	-	16.74 ± 9.14	-5.90	2.20	8.63	17.40	24.40	30.00	35.60
DTR (℃)	-	8.86 ± 4.29	0.60	2.30	5.50	8.70	11.70	16.20	22.10
RH (%)	-	76.29 ± 12.39	33.00	55.00	69.00	78.00	86.00	95.00	100.00
A P(hPa)	-	1012.11 ± 9.57	987.50	997.90	1003.80	1012.10	1019.48	1027.92	1041.00
WS (m/s)	-	2.03 ± 0.89	0.30	0.90	1.40	1.90	2.40	3.60	6.60
**Air pollutants**									
PM_2.5_ (μg/m^3^)	-	52.24 ± 33.82	5.00	16.00	29.00	44.00	66.00	117.00	351.00
PM_10_ (μg/m^3^)	-	81.61 ± 43.57	11.00	26.00	51.00	74.00	104.00	160.00	413.00
SO_2_ (μg/m^3^)	-	11.16 ± 6.91	2.00	3.00	6.00	10.00	14.00	25.00	58.00
NO_2_ (μg/m^3^)	-	40.27 ± 18.26	9.00	18.00	27.00	36.00	50.00	75.00	134.00
CO (μg/m^3^)	-	870.00 ± 310.00	320.00	490.00	650.00	800.00	1020.00	1460.00	2860.00
O_3_ (μg/m^3^)	-	56.48 ± 29.15	4.00	11.00	34.00	52.00	75.00	110.15	185.00

*SD* standard deviation, *T-mean* temperature mean, *DTR* diurnal temperature range, *AP* atmospheric pressure, *WS* wind speed, *PM2.5* particulate matter ≤ 2.5 μm in aerodynamic diameter, *PM10* particulate matter ≤ 10 μm in aerodynamic diameter, *SO*_*2*_ sulfur dioxide, *NO*_*2*_ nitrogen dioxide, *CO* carbon monoxide, *O*_*3*_ ozone

### Correlation analysis

**Fig. 2:**
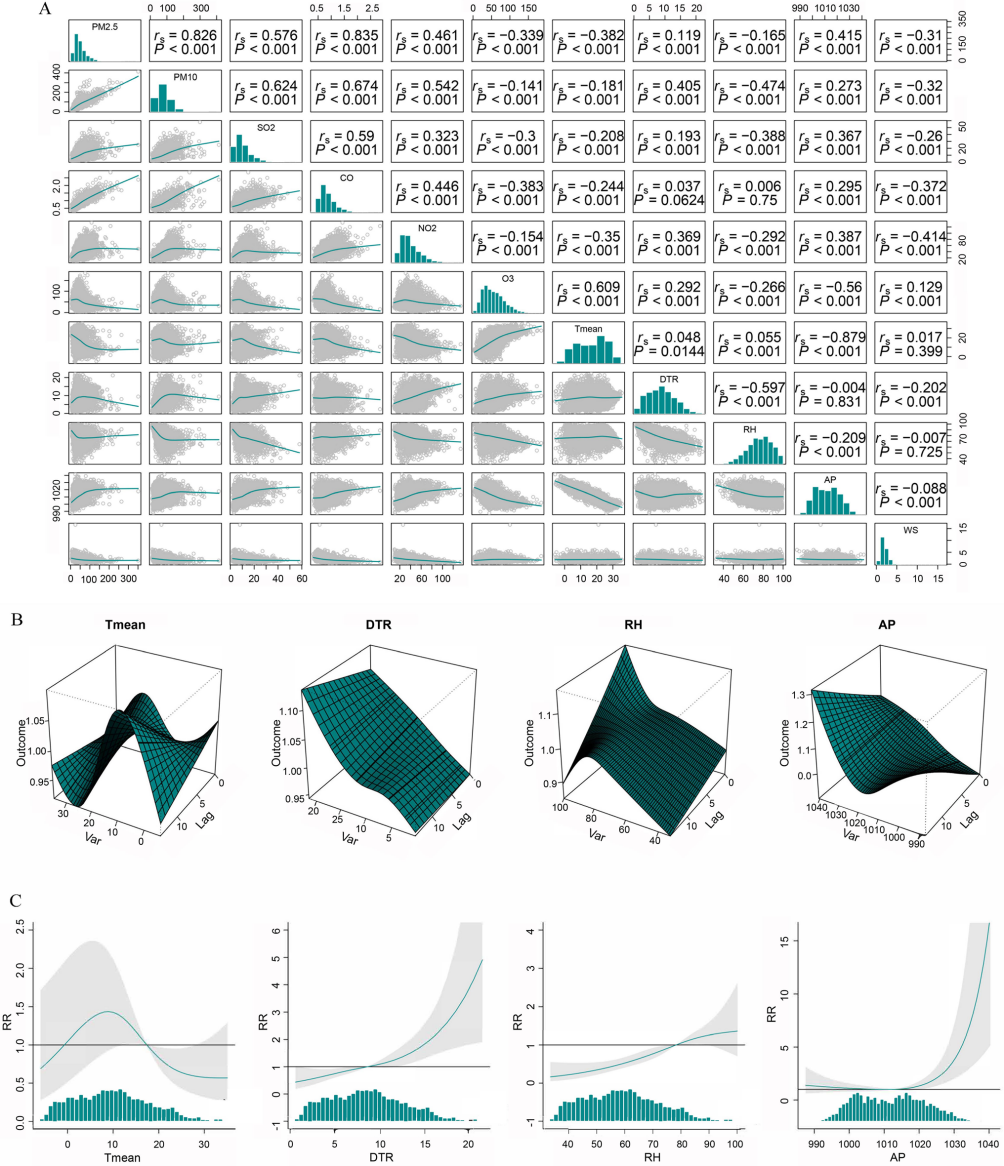
**A** Spearman’s correlation coefficients for meteorological factors and atmospheric pollutants. **B** A three-dimensional plot of the relative risk of daily SSNHL *vs*. T-mean, diurnal temperature range, relative humidity, and atmospheric pressure. **C** The overall exposure–response association with SSNHL. SSNHL, sudden sensorineural hearing loss

The outcomes of the Spearman correlation analysis of air pollutants and MF are shown in Fig. 2A. There were positive correlations between PM_2.5_ and PM_10_ (*P* < 0.001, *r*_*s*_ = 0.826), and between PM_2.5_ and CO (*P* < 0.001, *r*_*s*_ = 0.835). The T-mean and AP were negatively associated with NO_2_, PM_2.5_, SO_2_, PM_10_, and CO and positively associated with O_3_ (all *P* < 0.001). The DTR was positively associated with NO_2_, PM_2.5_, SO_2_, O_3_, PM_10_, and CO (all *P* < 0.001). However, RH was negatively associated with NO_2_, PM_2.5_, SO_2_, PM_10_, CO, and O_3_ (all *P* < 0.001).

### The overall effect of T-mean, DTR, RH, and AP on the admission rate for SSNHL

The exposure–response relationships of the T-mean, DTR, RH, and AP at different values with the admission rate for SSNHL are shown in Fig. 2B, C. We found a monotonically increasing trend between DTR and SSNHL hospitalization risk. Specifically, the risk of hospitalization in SSNHL increased with increasing DTR values. We also observed that low levels of AP were not associated with the risk of SSNHL admission, but after AP reached a certain level, AP values were positively associated with the risk of SSNHL admission. The association between the T-mean and the admission rate for SSNHL is shown in Table 2. In the single-day lag, with a median of 50.00% as the reference, extremely low temperature increased the risk of SSNHL admission from lag8 (RR = 1.032, 95% CI: 1.001–1.064) to lag14 (RR = 1.095, 95% CI: 1.03, 1.163), and lag14 had the highest RR. Table 3 records the relationship between DTR and the admission rate for SSNHL. Except for high and exceedingly high DTR, no significant effect was found for the DTR of other grades. Overall, extremely high DTR affected the admission rate for SSNHL on lag 0 day. The significance of the effect was greatest on that day (RR = 1.054, 95% CI: 1.007–1.104) and then gradually decreased. The cumulative risk curve showed a gradual increase in the cumulative risk. In terms of the high RH, the single-day lag association was statistically significant from lag0 to lag7 with the highest RR of SSNHL admission being at lag0 (RR = 1.029, 95%CI: 1.008, 1.050). In the cumulative lag structure, estimated risk effects appeared to be significant from lag0 to lag 0–14. Similar results were observed for the association between extremely high RH and the risk of SSNHL admission (Table 4). Interestingly, all grades of AP had significant effects on SSNHL (Table 5). The effects of low grades AP were delayed, whereas the effects of high grades AP were immediate.

**Table 2:** Relative risk (mean and 95% confidence intervals) of admission rate of sudden deafness for specific T-mean on different lag days

	Single-day lag					Cumulative-day lag			
Lag	5th percentile	25th percentile	75th percentile	95th percentile	Lag	5th percentile	25th percentile	75th percentile	95th percentile
0	0.966 (0.874, 1.067)	0.954 (0.895, 1.016)	1.029 (0.979, 1.083)	1.002 (0.925, 1.086)	0–0	0.966 (0.874, 1.067)	0.954 (0.895, 1.016)	1.029 (0.979, 1.083)	1.002 (0.925, 1.086)
1	0.972 (0.889, 1.062)	0.963 (0.901, 1.019)	1.021 (0.976, 1.068)	0.997 (0.928, 1.071)	0–1	0.938 (0.777, 1.133)	0.919 (0.815, 1.036)	1.051 (0.955, 1.157)	0.999 (0.858, 1.164)
2	0.978 (0.904, 1.057)	0.973 (0.925, 1.023)	1.013 (0.973, 1.055)	0.992 (0.930, 1.057)	0–2	0.917 (0.703, 1.197)	0.894 (0.754, 1.059)	1.065 (0.930, 1.220)	0.991 (0.798, 1.23)
3	0.984 (0.919, 1.053)	0.982 (0.940, 1.027)	1.005 (0.970, 1.042)	0.987 (0.932, 1.044)	0–3	0.902 (0.647, 1.259)	0.878 (0.709, 1.087)	1.070 (0.902, 1.270)	0.978 (0.745, 1.283)
4	0.990 (0.933, 1.049)	0.992 (0.954, 1.031)	0.997 (0.966, 1.029)	0.981 (0.933, 1.032)	0–4	0.893 (0.605, 1.318)	0.871 (0.678, 1.119)	1.067 (0.873, 1.305)	0.960 (0.697, 1.321)
5	0.996 (0.946, 1.048)	1.002 (0.968, 1.037)	0.989 (0.962, 1.018)	0.976 (0.933, 1.021)	0–5	0.889 (0.575, 1.376)	0.873 (0.658, 1.158)	1.056 (0.841, 1.325)	0.937 (0.653, 1.344)
6	1.002 (0.957, 1.049)	1.012 (0.981, 1.044)	0.981 (0.956, 1.007)	0.971 (0.932, 1.012)	0–6	0.891 (0.555, 1.432)	0.883 (0.648, 1.203)	1.036 (0.807, 1.330)	0.91 (0.612, 1.351)
7	1.008 (0.965, 1.053)	1.022 (0.992, 1.053)	0.974 (0.949, 0.999)	0.966 (0.928, 1.006)	0–7	0.899 (0.543, 1.487)	0.902 (0.648, 1.257)	1.009 (0.771, 1.320)	0.879 (0.574, 1.344)
8	1.014 (0.970, 1.061)	1.032 (1.001, 1.064)*	0.966 (0.941, 0.991)	0.961 (0.922, 1.002)	0–8	0.911 (0.538, 1.544)	0.931 (0.656, 1.321)	0.974 (0.733, 1.296)	0.844 (0.538, 1.325)
9	1.021 (0.971, 1.072)	1.042 (1.008, 1.077)*	0.958 (0.932, 0.985)	0.956 (0.913, 1.000)	0–9	0.930 (0.540, 1.604)	0.971 (0.673, 1.399)	0.934 (0.692, 1.260)	0.807 (0.503, 1.295)
10	1.027 (0.970, 1.087)	1.053 (1.014, 1.092)*	0.951 (0.922, 0.981)	0.951 (0.904, 1.000)	0–10	0.955 (0.546, 1.672)	1.022 (0.699, 1.493)	0.888 (0.65, 1.214)	0.767 (0.468, 1.257)
11	1.033 (0.968, 1.103)	1.063 (1.019, 1.109)*	0.943 (0.911, 0.977)	0.946 (0.893, 1.002)	0–11	0.987 (0.556, 1.753)	1.086 (0.733, 1.609)	0.837 (0.605, 1.160)	0.726 (0.433, 1.215)
12	1.040 (0.964, 1.121)	1.074 (1.023, 1.126)*	0.936 (0.900, 0.973)	0.941 (0.882, 1.004)	0–12	1.026 (0.568, 1.854)	1.166 (0.775, 1.754)	0.784 (0.557, 1.102)	0.683 (0.398, 1.171)
13	1.046 (0.960, 1.140)	1.084 (1.027, 1.144)*	0.928 (0.888, 0.970)	0.936 (0.870, 1.007)	0–13	1.073 (0.580, 1.986)	1.264 (0.824, 1.938)	0.728 (0.508, 1.041)	0.639 (0.362, 1.129)
14	1.052 (0.955, 1.160)	1.095 (1.03, 1.163)*	0.921 (0.877, 0.968)	0.931 (0.858, 1.010)	0–14	1.130 (0.590, 2.163)	1.384 (0.881, 2.173)	0.670 (0.458, 0.980)	0.595 (0.324, 1.091)

*T-mean* temperature mean

^*^P < 0.05

**Table 3:** Relative risk (mean and 95% confidence intervals) of admission rate of sudden deafness for specific DTR on different lag days

	Single-day lag					Cumulative-day lag			
Lag	5th percentile	25th percentile	75th percentile	95th percentile	Lag	5th percentile	25th percentile	75th percentile	95th percentile
0	0.955 (0.894, 1.020)	0.976 (0.954, 0.999)	1.023 (0.996, 1.051)	1.054 (1.007, 1.104)*	0–0	0.955 (0.894, 1.020)	0.976 (0.954, 0.999)	1.023 (0.996, 1.024)	1.054 (1.007, 1.104)*
1	0.956 (0.901, 1.014)	0.977 (0.958, 0.998)	1.022 (0.997, 1.047)	1.054 (1.011, 1.099)*	0–1	0.913 (0.806, 1.034)	0.954 (0.914, 0.996)	1.045 (0.993, 1.051)	1.111 (1.017, 1.213)*
2	0.957 (0.907, 1.009)	0.979 (0.961, 0.997)	1.020 (0.998, 1.043)	1.053 (1.014, 1.094)*	0–2	0.873 (0.731, 1.043)	0.934 (0.878, 0.993)	1.066 (0.991, 1.081)	1.170 (1.032, 1.327)*
3	0.957 (0.913, 1.004)	0.980 (0.964, 0.996)	1.019 (0.999, 1.040)	1.053 (1.018, 1.089)*	0–3	0.836 (0.668, 1.047)	0.915 (0.846, 0.989)	1.087 (0.991, 1.114)	1.232 (1.051, 1.445)*
4	0.958 (0.918, 1.000)	0.981 (0.966, 0.996)	1.018 (1.000, 1.036)*	1.053 (1.021, 1.086)*	0–4	0.801 (0.614, 1.046)	0.897 (0.818, 0.984)	1.106 (0.991, 1.152)	1.297 (1.074, 1.566)*
5	0.959 (0.922, 0.997)	0.982 (0.969, 0.996)	1.017 (1.000, 1.034)*	1.052 (1.023, 1.083)*	0–5	0.768 (0.567, 1.04)	0.881 (0.794, 0.979)	1.125 (0.993, 1.195)	1.365 (1.101, 1.692)*
6	0.960 (0.925, 0.996)	0.983 (0.971, 0.996)	1.016 (1.000, 1.031)*	1.052 (1.024, 1.080)*	0–6	0.737 (0.527, 1.031)	0.866 (0.772, 0.973)	1.142 (0.995, 1.245)	1.436 (1.132, 1.822)*
7	0.960 (0.926, 0.996)	0.984 (0.972, 0.997)	1.014 (0.999, 1.030)	1.052 (1.025, 1.079)*	0–7	0.708 (0.492, 1.018)	0.853 (0.752, 0.967)	1.159 (0.997, 1.302)	1.510 (1.166, 1.956)*
8	0.961 (0.926, 0.997)	0.985 (0.973, 0.998)	1.013 (0.998, 1.029)	1.051 (1.024, 1.080)*	0–8	0.680 (0.462, 1.003)	0.840 (0.735, 0.962)	1.174 (1.000, 1.369)*	1.588 (1.204, 2.095)*
9	0.962 (0.925, 1.000)	0.987 (0.973, 1.000)	1.012 (0.996, 1.029)	1.051 (1.022, 1.081)*	0–9	0.655 (0.434, 0.988)	0.829 (0.719, 0.956)	1.188 (1.002, 1.449)*	1.669 (1.243, 2.240)*
10	0.963 (0.922, 1.005)	0.988 (0.973, 1.003)	1.011 (0.993, 1.029)	1.051 (1.019, 1.083)*	0–10	0.630 (0.408, 0.972)	0.819 (0.705, 0.952)	1.201 (1.003, 1.546)*	1.753 (1.284, 2.393)*
11	0.963 (0.919, 1.010)	0.989 (0.973, 1.005)	1.010 (0.99, 1.030)	1.050 (1.015, 1.087)*	0–11	0.607 (0.385, 0.958)	0.810 (0.691, 0.949)	1.212 (1.003, 1.665)*	1.841 (1.326, 2.557)*
12	0.964 (0.915, 1.016)	0.990 (0.972, 1.008)	1.008 (0.987, 1.031)	1.050 (1.011, 1.090)*	0–12	0.585 (0.362, 0.946)	0.802 (0.679, 0.948)	1.222 (1.001, 1.814)*	1.933 (1.367, 2.734)*
13	0.965 (0.910, 1.023)	0.991 (0.971, 1.012)	1.007 (0.983, 1.032)	1.049 (1.006, 1.094)*	0–13	0.565 (0.340, 0.939)	0.795 (0.666, 0.949)	1.231 (0.996, 2.003)	2.029 (1.405, 2.930)*
14	0.966 (0.905, 1.031)	0.992 (0.970, 1.015)	1.006 (0.980, 1.033)	1.049 (1.002, 1.099)*	0–14	0.545 (0.318, 0.936)	0.789 (0.654, 0.953)	1.239 (0.989, 2.247)	2.128 (1.438, 3.149)*

*DTR* diurnal temperature range

^*^P < 0.05

**Table 4:** Relative risk (mean and 95% confidence intervals) of admission rate of sudden deafness for specific RH on different lag days

	Single-day lag					Cumulative-day lag			
Lag	5th percentile	25th percentile	75th percentile	95th percentile	lag	5th percentile	25th percentile	75th percentile	95th percentile
0	0.973 (0.933, 1.013)	0.992 (0.968, 1.016)	1.029 (1.008, 1.050)*	1.104 (1.044, 1.168)*	0–0	0.973 (0.933, 1.013)	0.992 (0.968, 1.016)	1.029 (1.008, 1.050)*	1.104 (1.044, 1.168)*
1	0.969 (0.934, 1.005)	0.99 (0.969, 1.012)	1.027 (1.008, 1.045)*	1.092 (1.038, 1.148)*	0–1	0.942 (0.872, 1.019)	0.982 (0.937, 1.028)	1.056 (1.016, 1.098)*	1.206 (1.084, 1.341)*
2	0.965 (0.934, 0.998)	0.988 (0.969, 1.008)	1.024 (1.008, 1.041)*	1.079 (1.032, 1.128)*	0–2	0.909 (0.814, 1.016)	0.970 (0.909, 1.036)	1.082 (1.024, 1.142)*	1.301 (1.118, 1.513)*
3	0.961 (0.934, 0.990)	0.987 (0.970, 1.004)	1.022 (1.007, 1.037)*	1.066 (1.025, 1.109)*	0–3	0.874 (0.760, 1.006)	0.957 (0.881, 1.040)	1.105 (1.032, 1.184)*	1.387 (1.147, 1.677)*
4	0.958 (0.933, 0.983)	0.985 (0.970, 1.001)	1.019 (1.006, 1.033)*	1.054 (1.018, 1.092)*	0–4	0.837 (0.710, 0.988)	0.943 (0.855, 1.040)	1.126 (1.039, 1.222)*	1.462 (1.169, 1.829)*
5	0.954 (0.932, 0.977)	0.984 (0.970, 0.998)	1.017 (1.005, 1.029)*	1.042 (1.010, 1.075)*	0–5	0.799 (0.663, 0.964)	0.927 (0.830, 1.036)	1.145 (1.045, 1.256)*	1.523 (1.183, 1.961)*
6	0.951 (0.930, 0.972)	0.982 (0.969, 0.995)	1.015 (1.004, 1.026)*	1.030 (1.000, 1.060)*	0–6	0.759 (0.618, 0.933)	0.911 (0.806, 1.029)	1.162 (1.050, 1.286)*	1.568 (1.189, 2.069)*
7	0.947 (0.927, 0.967)	0.98 (0.968, 0.993)	1.012 (1.002, 1.023)*	1.018 (0.989, 1.047)	0–7	0.719 (0.576, 0.898)	0.893 (0.782, 1.019)	1.176 (1.055, 1.312)*	1.596 (1.185, 2.150)*
8	0.943 (0.923, 0.964)	0.979 (0.966, 0.992)	1.01 (0.999, 1.021)	1.006 (0.977, 1.036)	0–8	0.678 (0.535, 0.859)	0.874 (0.759, 1.006)	1.188 (1.058, 1.334)*	1.606 (1.171, 2.201)*
9	0.940 (0.918, 0.961)	0.977 (0.963, 0.991)	1.007 (0.996, 1.019)	0.994 (0.963, 1.026)	0–9	0.637 (0.497, 0.818)	0.854 (0.736, 0.991)	1.196 (1.059, 1.352)*	1.597 (1.147, 2.223)*
10	0.936 (0.913, 0.960)	0.976 (0.961, 0.991)	1.005 (0.992, 1.018)	0.983 (0.949, 1.018)	0–10	0.597 (0.460, 0.774)	0.833 (0.713, 0.974)	1.202 (1.058, 1.367)*	1.569 (1.110, 2.217)*
11	0.933 (0.907, 0.959)	0.974 (0.957, 0.991)	1.003 (0.989, 1.017)	0.971 (0.933, 1.011)	0–11	0.556 (0.424, 0.731)	0.812 (0.689, 0.956)	1.206 (1.055, 1.378)*	1.524 (1.063, 2.186)*
12	0.929 (0.900, 0.959)	0.972 (0.954, 0.992)	1.000 (0.985, 1.016)	0.960 (0.918, 1.004)	0–12	0.517 (0.389, 0.687)	0.789 (0.664, 0.937)	1.206 (1.048, 1.388)*	1.463 (1.003, 2.135)*
13	0.925 (0.893, 0.959)	0.971 (0.950, 0.992)	0.998 (0.980, 1.016)	0.949 (0.902, 0.998)	0–13	0.478 (0.355, 0.645)	0.766 (0.639, 0.919)	1.204 (1.038, 1.395)*	1.389 (0.932, 2.068)
14	0.922 (0.886, 0.959)	0.969 (0.946, 0.993)	0.996 (0.976, 1.016)	0.938 (0.886, 0.993)	0–14	0.441 (0.321, 0.606)	0.743 (0.612, 0.901)	1.198 (1.024, 1.402)*	1.303 (0.852, 1.990)

*RH* relative humidity

^*^P < 0.05

**Table 5:** Relative risk (mean and 95% confidence intervals) of admission rate of sudden deafness for specific AP on different lag days

	Single-day lag					Cumulative-day lag			
Lag	5th percentile	25th percentile	75th percentile	95th percentile	Lag	5th percentile	25th percentile	75th percentile	95th percentile
0	0.931 (0.878, 0.986)	0.954 (0.919, 0.991)	1.042 (1.007, 1.079)*	1.078 (1.007, 1.154)*	0–0	0.931 (0.878, 0.986)	0.954 (0.919, 0.991)	1.042 (1.007, 1.079)*	1.078 (1.007, 1.154)*
1	0.942 (0.894, 0.992)	0.961 (0.929, 0.994)	1.039 (1.007, 1.071)*	1.077 (1.013, 1.145)*	0–1	0.877 (0.785, 0.978)	0.917 (0.854, 0.985)	1.083 (1.015, 1.156)*	1.161 (1.021, 1.321)*
2	0.953 (0.91, 0.997)	0.968 (0.94, 0.997)	1.035 (1.007, 1.064)*	1.077 (1.019, 1.137)*	0–2	0.835 (0.715, 0.975)	0.888 (0.803, 0.982)	1.121 (1.022, 1.229)*	1.25 (1.041, 1.502)*
3	0.964 (0.926, 1.003)	0.975 (0.95, 1.001)	1.031 (1.006, 1.057)*	1.076 (1.024, 1.130)*	0–3	0.805 (0.662, 0.978)	0.865 (0.762, 0.982)	1.156 (1.029, 1.299)*	1.345 (1.067, 1.696)*
4	0.975 (0.941, 1.01)	0.982 (0.96, 1.005)	1.028 (1.005, 1.050)*	1.075 (1.029, 1.123)*	0–4	0.785 (0.624, 0.986)	0.85 (0.732, 0.987)	1.188 (1.035, 1.363)*	1.446 (1.100, 1.902)*
5	0.986 (0.956, 1.017)	0.989 (0.969, 1.01)	1.024 (1.004, 1.044)*	1.074 (1.033, 1.118)*	0–5	0.774 (0.598, 1.001)	0.841 (0.711, 0.995)	1.217 (1.041, 1.422)*	1.553 (1.139, 2.119)*
6	0.998 (0.97, 1.026)	0.996 (0.978, 1.015)	1.020 (1.002, 1.039)*	1.074 (1.035, 1.114)*	0–6	0.772 (0.583, 1.023)	0.838 (0.697, 1.007)	1.241 (1.046, 1.474)*	1.668 (1.184, 2.349)*
7	1.009 (0.983, 1.036)	1.004 (0.986, 1.022)	1.017 (0.999, 1.035)	1.073 (1.035, 1.112)*	0–7	0.779 (0.577, 1.053)	0.841 (0.690, 1.024)	1.262 (1.049, 1.519)*	1.789 (1.236, 2.590)*
8	1.021 (0.994, 1.049)	1.011 (0.992, 1.03)	1.013 (0.995, 1.032)	1.072 (1.034, 1.112)*	0–8	0.796 (0.580, 1.092)	0.85 (0.690, 1.047)	1.278 (1.050, 1.557)*	1.919 (1.294, 2.844)*
9	1.033 (1.003, 1.064)*	1.018 (0.998, 1.038)	1.009 (0.990, 1.029)	1.071 (1.030, 1.114)*	0–9	0.822 (0.592, 1.142)	0.865 (0.696, 1.076)	1.291 (1.049, 1.588)*	2.056 (1.357, 3.114)*
10	1.045 (1.011, 1.08)*	1.026 (1.003, 1.048)*	1.006 (0.984, 1.028)	1.071 (1.026, 1.118)*	0–10	0.859 (0.612, 1.207)	0.887 (0.708, 1.112)	1.298 (1.044, 1.613)*	2.201 (1.423, 3.404)*
11	1.057 (1.018, 1.098)*	1.033 (1.007, 1.059)*	1.002 (0.978, 1.027)	1.070 (1.020, 1.123)*	0–11	0.909 (0.640, 1.290)	0.917 (0.725, 1.159)	1.301 (1.036, 1.634)*	2.355 (1.491, 3.719)*
12	1.07 (1.025, 1.116)*	1.04 (1.011, 1.071)*	0.999 (0.972, 1.026)	1.069 (1.013, 1.128)*	0–12	0.972 (0.677, 1.396)	0.954 (0.748, 1.217)	1.299 (1.023, 1.650)*	2.518 (1.559, 4.067)*
13	1.082 (1.031, 1.136)*	1.048 (1.015, 1.082)*	0.995 (0.965, 1.026)	1.069 (1.006, 1.135)*	0–13	1.052 (0.721, 1.533)	0.999 (0.775, 1.289)	1.293 (1.005, 1.663)*	2.691 (1.622, 4.462)*
14	1.095 (1.036, 1.156)*	1.056 (1.018, 1.094)*	0.992 (0.959, 1.026)	1.068 (0.999, 1.142)	0–14	1.151 (0.774, 1.712)	1.055 (0.806, 1.381)	1.282 (0.981, 1.675)*	2.873 (1.679, 4.917)*

*AP* atmospheric pressure

^*^P < 0.05

### Stratified analysis by age and sex

**Fig. 3:**
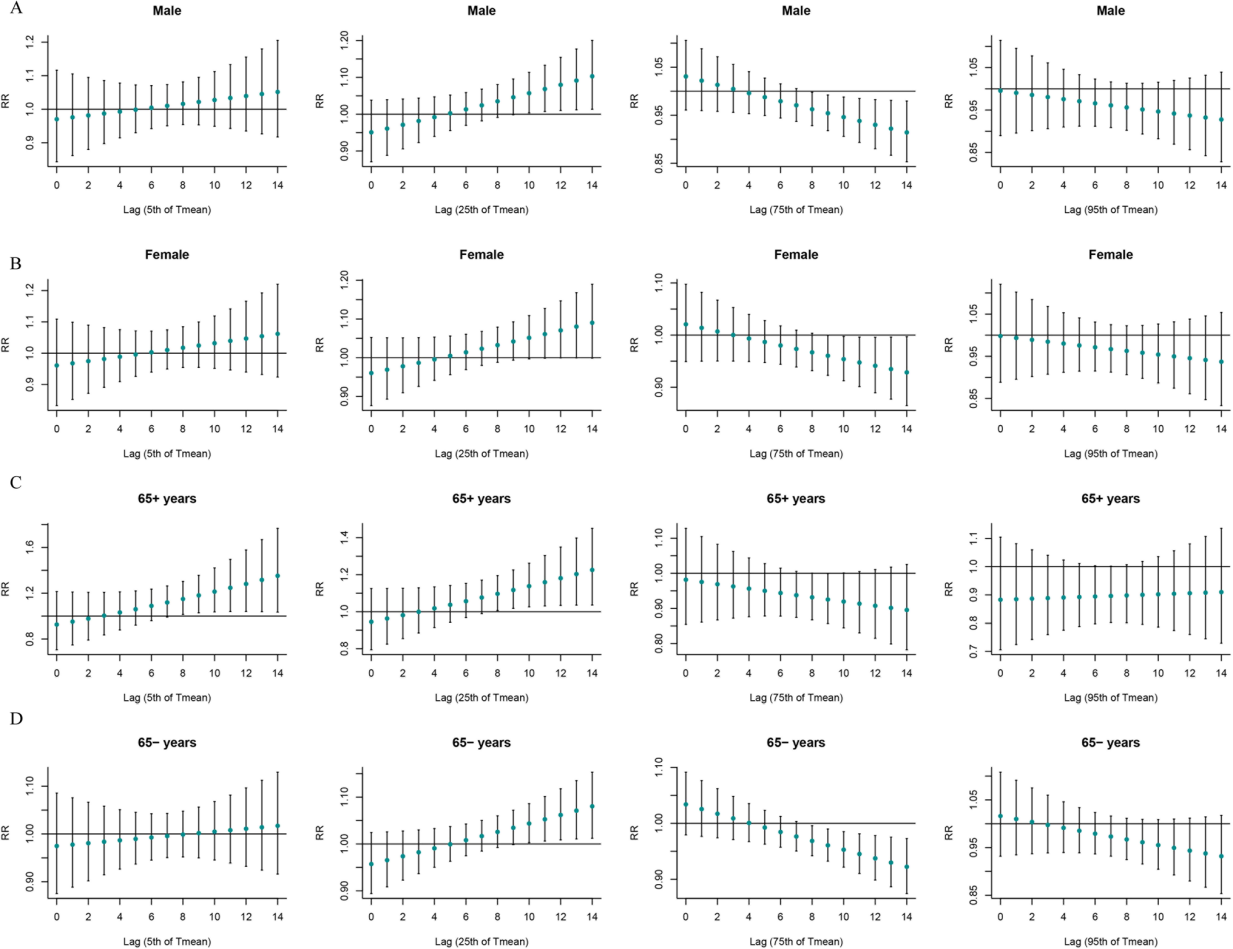
Single-day lag effect estimates in SSNHL in T-mean stratified by age and sex. SSNHL, sudden sensorineural hearing loss

**Fig. 4:**
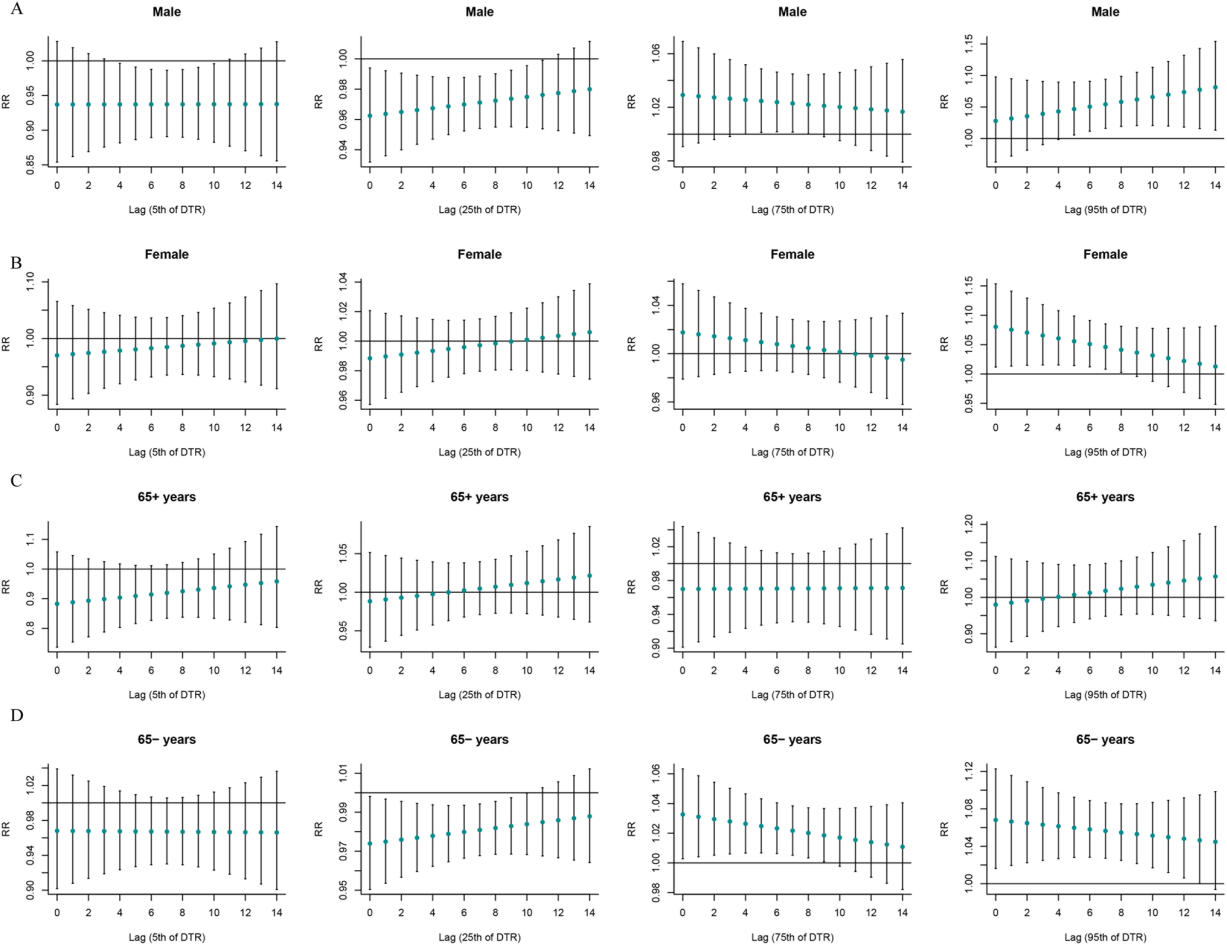
Single-day lag effect estimates in SSNHL in diurnal temperature range stratified by age and sex. SSNHL, sudden sensorineural hearing loss

**Fig. 5:**
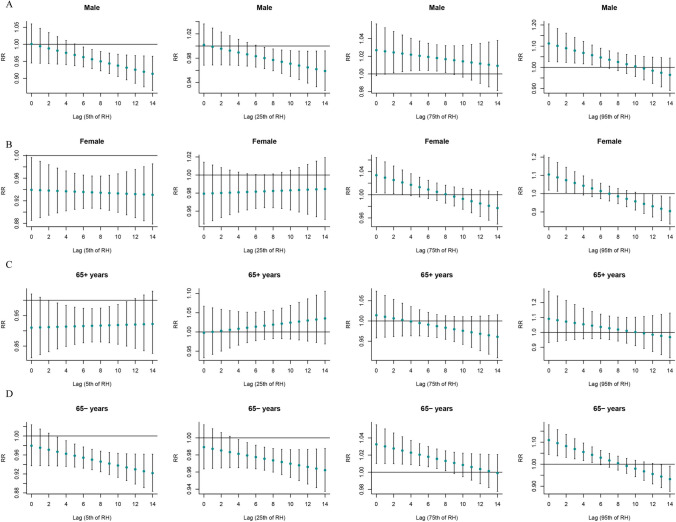
Single-day lag effect estimates in SSNHL in relative humidity stratified by age and sex. SSNHL, sudden sensorineural hearing loss

**Fig. 6:**
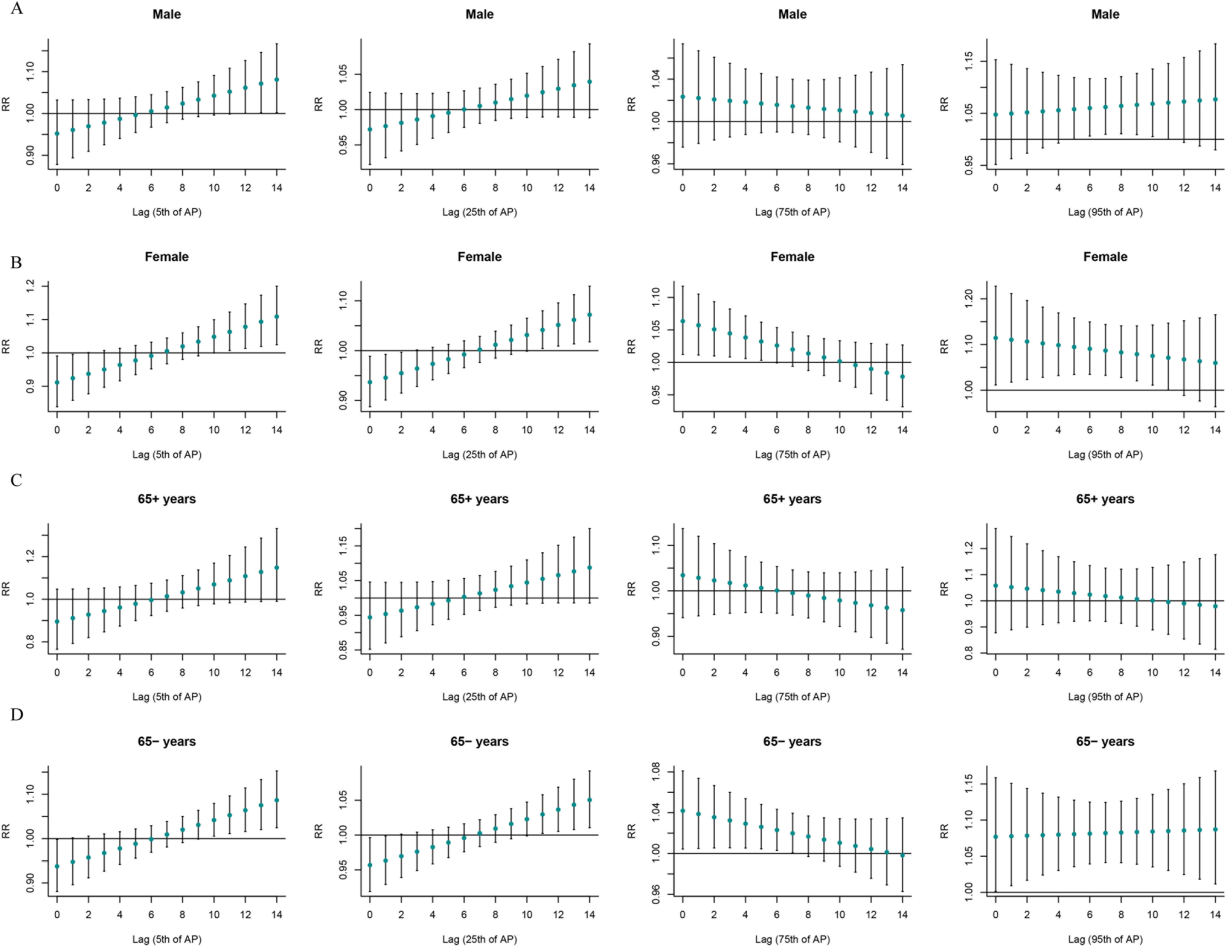
Single-day lag effect estimates in SSNHL in atmospheric pressure stratified by age and sex. SSNHL, sudden sensorineural hearing loss

Figure 3 shows the stratified analysis results of the “”T-mean according to age and sex. Both hypothermia and hyperthermia had significant effects on SSNHL. At very low DTR, men were affected, and the effect appeared on day 4 of the delay and lasted for 7 days. Both men and women were affected by extremely high DTR, and the effects on women were immediate, whereas men showed significant effects only after a delay of 5 days (Fig. 4). At very low RH, men and women as well as the elderly and non-elderly were affected. At extremely high RH, all but the older individuals were affected (Fig. 5). Among older individuals and men, SSNHL was almost unaffected by AP. However, under different AP levels, non-elderly women were affected, and the effects were delayed and sustained to different degrees (Fig. 6).

### Literature review

Using the originally set search terms, we further read the full text after reviewing the titles, abstracts, and eliminating duplicates, resulting in the inclusion of 15 literatures (Herbert et al. 1987; Preyer 1996; Danielides et al. 2002; Lin et al. 2006; Li and Feng 2013; Seo et al. 2014; Yun et al. 2014; Ryu et al. 2017; Lee et al. 2019a; Choi et al. 2019; Zhang et al. 2019b; Tsai et al. 2021; Zhang et al. 2021a; Tang et al. 2022; Cheng et al. 2022). We deeply investigate the possible effects and mechanisms of meteorological factors on SSNHL from several aspects: wind speed, air pressure, temperature, humidity, and air pollutants (Table 6). Database and search strategy are shown in Supplement Table 1.

**Table 6:** Results of studies reporting the influence of weather condition on the occurrence of ISSHL

Association of weather and SSNHL	Authors	Ref	Year	Country	No. of patients	Study period (year)	Study design	Conclusion
Air pollution	Hyun Min Lee	Lee et al. (2019a)	2019	Korea	817	1	Retrospective observational study	PM2.5 maximums were weakly negatively correlated with SSNHL admissions
	Hyo Geun Choi	Choi et al. (2019)	2019	Korea	5200	12	Retrospective observational study	SSNHL is associated with high levels of NO2
	S. C. Tsai	Tsai et al. (2021)	2021	China	64321	13	Cross-sectional study	Exposure to high levels of CO, NO, NO2 and PM2.5 significantly increased the risk of SSNHL
	S. E. Tang	Tang et al. (2022)	2022	China	12497	16	Retrospective observational study	SSNHL is associated with high levels of PM2.5
	C. G. Cheng	Cheng et al. (2022)	2022	China	850	9	Time-series study	Air pollutants were influential factors of SSNHL, with the lag effect
Temperature	S. Preyer	Preyer (1996)	1996	NS	128	1	Prospective study	Patients with fully restored hearing thresholds had smaller values of temperature change compared with those whose hearing was not restored
	Kejun Li	Li and Feng (2013)	2013	China	NS	29	Retrospective observational study	SSNHL is associated with temperature
	Hyun Min Lee	Lee et al. (2019a)	2019	Korea	817	1	Retrospective observational study	Temperature was negatively correlated with SSNHL
	Jilei Zhang	Zhang et al. (2019b)	2019	China	175	8	Retrospective observational study	Association between onset of different subtypes of SSNHL and the temperature of the day
Atmospheric pressure	I. Herbert	Herbert et al. (1987)	1987	German	NS	NS	Retrospective observational study	SSNHL is associated with low levels of pressure
	S. Preyer	Preyer (1996)	1996	NS	128	1	Prospective study	Patients with fully restored hearing thresholds had lower values of pressure compared with those whose hearing was not restored
	Hyun Min Lee	Lee et al. (2019a)	2019	Korea	817	1	Retrospective observational study	Weak positive correlation between daily atmospheric pressure range and number of SSNHL patients
Wind speed	Jae-Hyun Seo	Seo et al. (2014)	2014	Korea	607	6	Retrospective observational study	SSNHL is associated with strong wind speed
	C. J. Yun	Yun et al. (2014)	2014	Korea	NS	NS	Retrospective observational study	SSNHL is associated with strong wind speed
	Hyun Min Lee	Lee et al. (2019a)	2019	Korea	817	1	Retrospective observational study	SSNHL is associated with strong wind speed
NS	V. Danielides	Danielides et al. (2002)	2002	Greece	82	5	Retrospective Observational Study	There is no support for meteorological factors affecting SSNHL
	Herng-Ching Lin	Lin et al. (2006)	2006	China	8712	5	Retrospective observational study	There is no support for meteorological factors affecting SSNHL
	In Yong Ryu	Ryu et al. (2017)	2017	Korea	318	11	Retrospective observational study	There is no support for meteorological factors affecting SSNHL
Daily temperature range	C. J. Yun	Yun et al. (2014)	2014	Korea	NS	NS	Retrospective observational study	SSNHL is associated with wide temperature differences
	Jilei Zhang	Zhang et al. (2021a)	2021	China	510	12	Cross-sectional study	Audiogram patterns of SSNHL decline correlate with wider diurnal temperature ranges

*SD* standard deviation, *T-mean* temperature mean, *DTR* diurnal temperature range, *AP* atmospheric pressure, *WS* wind speed, *PM2.5* particulate matter ≤ 2.5 μm in aerodynamic diameter, *PM10* particulate matter ≤ 10 μm in aerodynamic diameter, *SO*_*2*_ sulfur dioxide, *NO*_*2*_ nitrogen dioxide, *CO* carbon monoxide, *O*_*3*_ ozone

## Discussion

Hippocrates proposed that meteorological changes could affect human health in the fifth century (Nastos and Matzarakis 2006). And the impact of meteorological factors on acute gouty arthritis (Park et al. 2017), rheumatoid arthritis (Azzouzi and Ichchou 2020), systemic lupus erythematosus, and Behcet’s disease (Lee et al. 2015) has now been demonstrated. As a common emergency in otorhinolaryngology, the etiology of SSNHL is not clear. Now, although there are many studies reporting the seasonal and meteorological effects on the incidence, severity, and prognosis of SSNHL, controversy remains. So, we collated the previous relevant literature to deeply investigate the possible effects and mechanisms of meteorological factors on SSNHL from several aspects: wind speed, air pressure, temperature, humidity, and air pollutants. In addition, a time-series analysis integrating distributed lag non-linear models and generalized linear models was used in order to investigate the short-term associations between SSNHL patients admitted to the hospital and Hefei climatic variables.

### Wind speed and atmospheric pressure

**Fig. 7:**
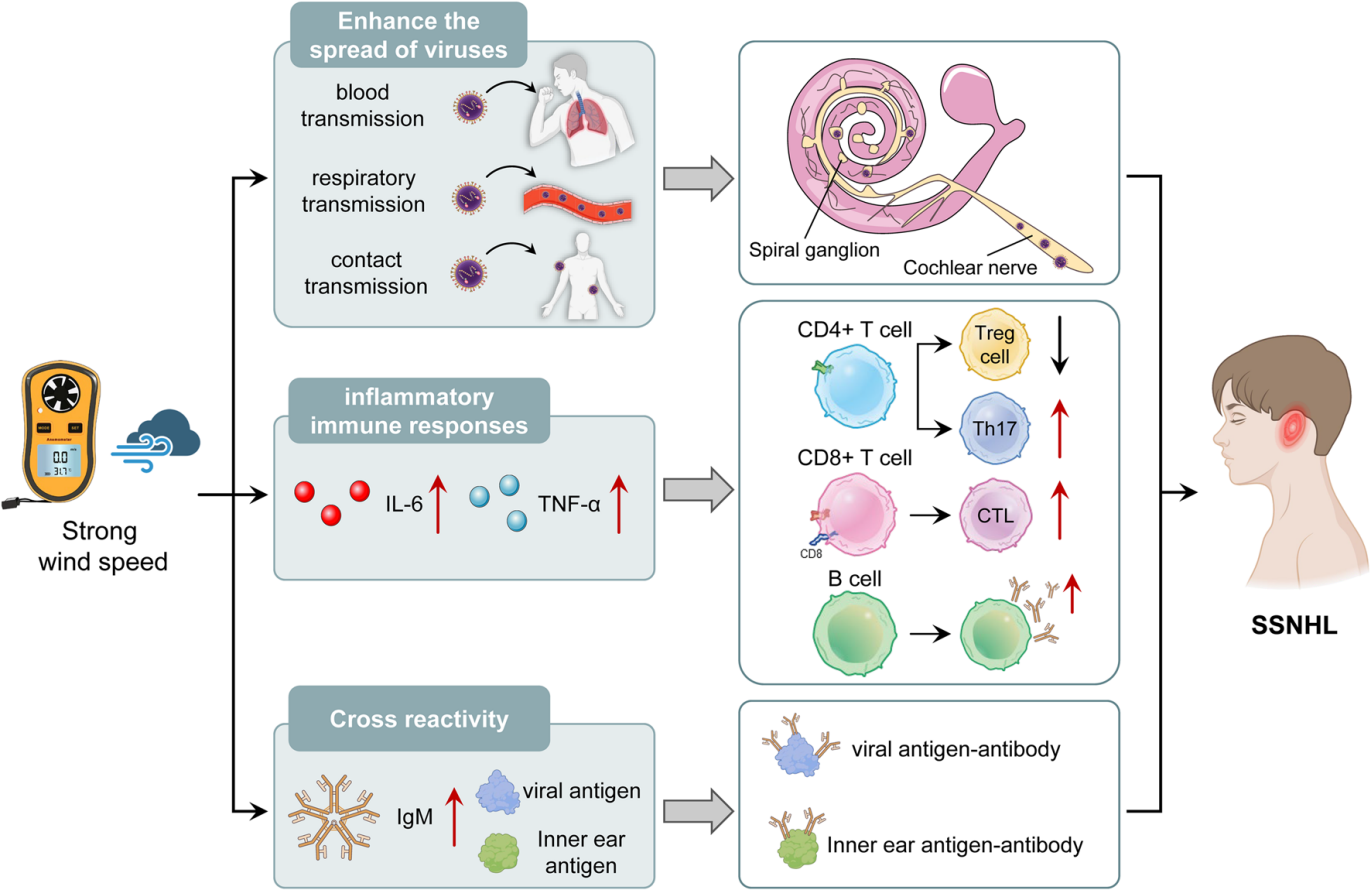
Potential mechanisms for linkage of wind speed and SSNHL. Strong wind speed can trigger SSNHL from three pathways: enhanced viral transmission, inflammatory immune response, and cross-reactivity. Enhanced viral transmission: In the case of strong wind speed, the virus can invade the spiral ganglion through respiratory transmission and blood transmission, infect the cochlear nerve, and cause hearing damage; inflammatory immune response: strong wind speed can cause elevated levels of IL-6 and TNF and promote the activation and differentiation of CD4T cells, CD8T cells, and B cells, thereby triggering inflammatory responses; cross-reactivity: strong wind speed can trigger cross-reactions and promote IgM production, which binds virus antigens and accidentally binds inner ear antigens and damaging the inner ear. SSNHL, sudden sensorineural hearing loss; IL-6, interleukin 6; TNF, tumor necrosis factor; NF-κB, nuclear factor kappa-B; SIP, steroid receptor coactivator, SRC, SRC-interacting protein; IgM, ImmunoglobulinM; Th17, T helper cell 17; CTL, cytotoxic T lymphocyte; CD4 + , cluster of differentiation 4 plus; CD8 + cluster of differentiation 8 plus

Recent viral infection is known to be associated with SSNHL. Several possible mechanisms have been proposed to explain how viral infections lead to SSNHL. The first may be that viral invasion of the cochlear nerve and spiral ganglia through the bloodstream or other routes induces SSNHL (Merchant et al. 2008); the second is that under certain conditions, viral reactivation in inner ear tissues initiates the inner ear’s immune response system, activating multiple inflammatory factors, causing endotheliosis and cytokine activation, which can lead to macrothrombi in the inner ear; the third is that viral infection triggers antigenic cross-reactivity in the inner ear, damaging the inner ear (Wilson 1986). Seo et al. reported that SSNHL incidence was significantly associated with mean wind speed and maximum wind speed when investigating the association between meteorological factors and SSNHL (Seo et al. 2014). Lee (Lee et al. 2019a) and Yun’s (Yun et al. 2014) findings also supported this view, which may be because strong winds increase the spread of the virus and enhance viral susceptibility (Fig. 7). Moreover, viral reactivation is based on decreased immune activity, which can be triggered by metabolic changes, physiological stressors, co-infections, cold, psychological stressors, and immunosuppressive states. So strong wind may induce the reactivation of neurotropic virus in spiral ganglion by decreasing immune activity and increase the risk of SSNHL (Seo et al. 2014). It has already been reported that higher wind speed may cause idiopathic facial paralysis and vestibular neuritis, and its etiology is similar to that of SSNHL, supporting the association between wind speed and the pathogenesis of SSNHL (Jeon et al. 2013).

**Fig. 8:**
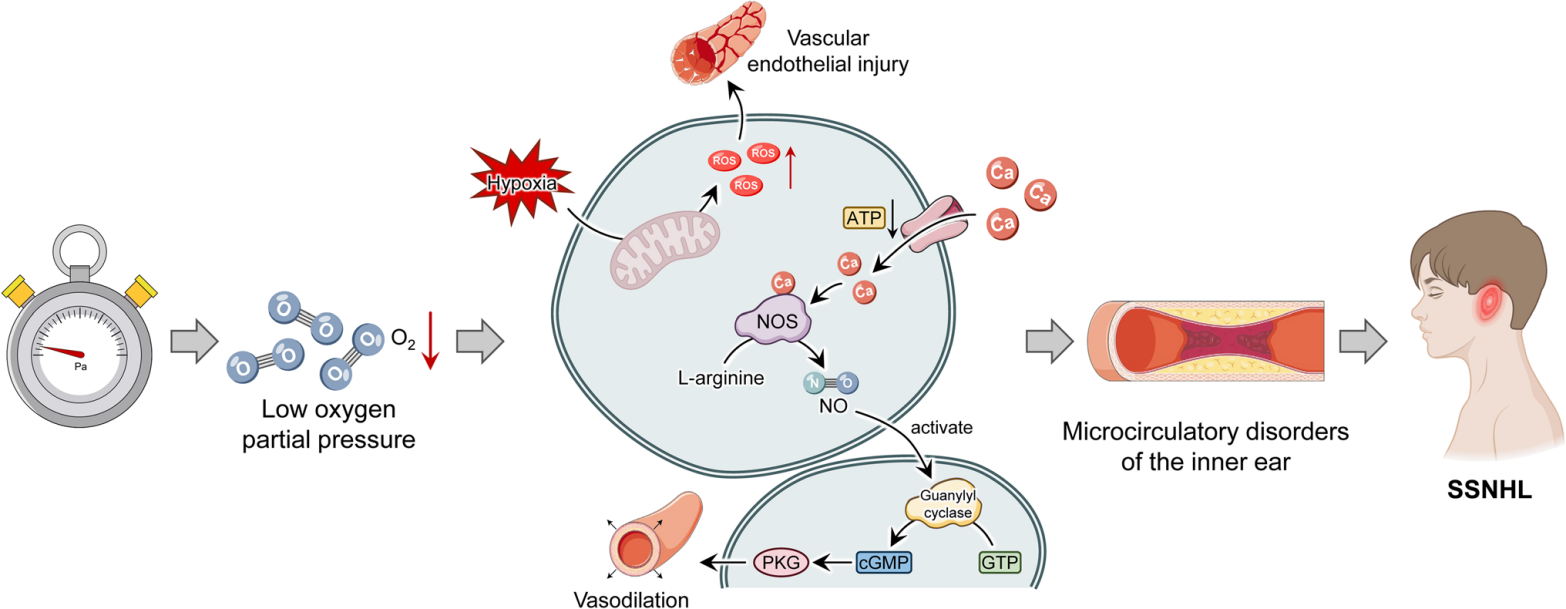
Potential mechanisms for linkage of atmospheric pressure and SSNHL. Under the conditions of low air pressure, the partial pressure of oxygen decreases, and hypoxia-induced ROS. ROS depresses levels of NO, induces monocyte invasion, elevates lipid peroxidation, promotes phenotype switching of VSMCs, induces EC dysfunction, precipitates inflammation as well as alters vascular responses and vasotone, which can cause vascular endothelial damage on the one hand, and on the other hand, it can promote Ca^2+^ inward flow, consume ATP, inhibit the guanylate cycle, reduce cGMP production, and cause vascular smooth muscle spasm, resulting in microcirculation disorders in the inner ear. SSNHL, sudden sensorineural hearing loss; ROS, reactive oxygen species; ATP, adenosine triphosphate; cGMP, cyclic guanosine monophosphate; O_2_, oxygen; NO, nitrogen; GTP, guanosine triphosphate; PKG, protein kinase G

An increase in tympanic membrane tension, which, in turn, results from a variance between middle ear pressure and AP, is thought to be the origin of a feeling of ear fullness in normal ears (Sakata et al. 2009). Comprehensive focus is drawn to the processes behind the feeling of ear fullness in acute sensorineural hearing loss, including Meniere’s disease, acute low-tone sensorineural hearing loss, abrupt deafness, and additional hearing-related illnesses. According to Sakata et al. (2012), there was a noticeable disparity in the minimum sensory threshold for air pressure with negative pressure between the affected and unaffected side of SSNHL patients, suggesting AP to be a key factor in the onset of SSNHL. Herbert suggests that SSNHL is more likely to occur at low pressure, especially when the pressure difference is large (Herbert et al. 1987), which is also supported by Preyer (1996). And he compared patients with complete recovery of hearing thresholds with those who had not yet recovered hearing and found that the former had smaller differences in air pressure and air temperature changes. We hypothesized that this may be due to the lower partial pressure of oxygen during hypobaric pressure, which leads to impaired microcirculation in the inner ear by affecting the production of reactive oxygen species (ROS) and related pathways (Fig. 8) (Alde et al. 2023). In addition, hyperbaric oxygen treatment is considered a salvage therapy to treat SSNHL, which indicates the effect of AP on SSNHL. However, there is no consensus on the relationship between air pressure and SSNHL, and further verification and discussion are still needed.

In contrast to previous studies, our results indicate that all grades of AP have a significant effect on SSNHL. The effects of low grades AP were delayed, whereas the effects of high grades AP were immediate. Besides, we found that AP affected only young women, but not the rest of the population. Given the lack of relevant research in this context, we conjecture that estrogen might play an important role and explain the differences between individuals with and without sensitivity to AP.

### Temperature and humidity

The current study with a time-series analysis examined whether the subpopulation-specific (stratified by sex, age, and status of hospital admission) correlations between MF such as T-mean, DTR, AP, and RH, and hospital outpatients for SSNHL varied over the short term and were non-linear and lag-related. We discovered that there was an enhanced positive correlation between DTR and SSNHL risk. This implies that vascular factors may contribute to the development of this subtype of SSNHL. At present, many studies have confirmed the association between cerebrovascular disease and SSNHL. Lin et al. (2008) found that patients with SSNHL had a significantly higher risk of stroke within 5 years than controls, and stroke patients also had a significantly higher probability of developing SSNHL than non-stroke patients (Kuo et al. 2016). For cerebrovascular disease, acute attack of cerebrovascular disease is closely related to temperature, and rapid changes in environmental temperature will dramatically increase the risk of stroke (Lavados et al. 2018; Mohammad et al. 2018). This suggests that ambient temperature may cause the onset of SSNHL.

**Fig. 9:**
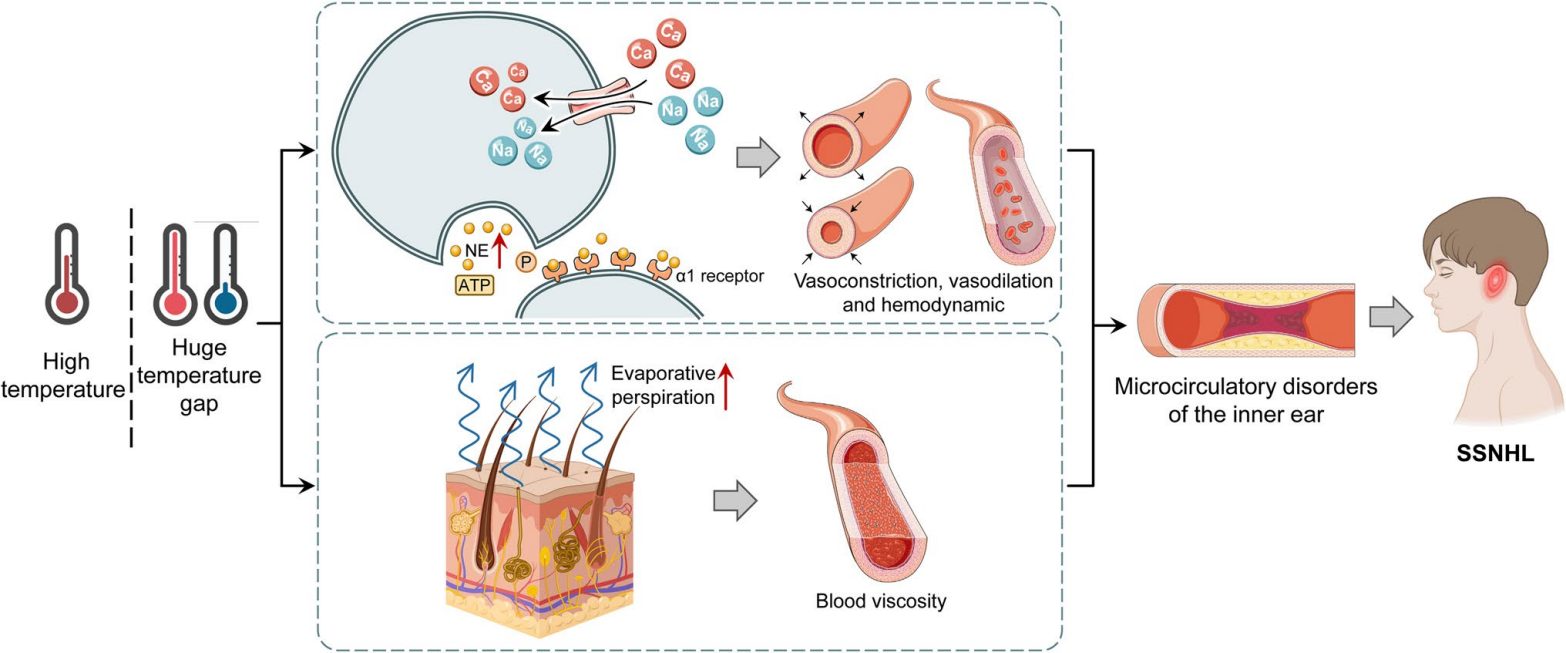
Potential mechanisms for linkage of temperature and SSNHL. Under high temperatures or large temperature differences, on the one hand, norepinephrine is elevated, which promotes vascular smooth muscle contraction by binding to α1 receptors. Changes in blood flow within the inner ear lead to microcirculatory disorders, causing ischemia, decreased blood oxygen levels, and edema in local tissue cells of the inner ear. This ultimately results in damage to the nerves of the inner ear; on the other hand, high-temperature conditions cause microcirculatory disorders in the inner ear due to the diastole of erector spinae muscles and blood vessels, the increased secretion of sweat, and the increased viscosity of blood, which lead to microcirculatory disorders and damage of the inner ear. SSNHL, sudden sensorineural hearing loss; ATP, adenosine triphosphate; NE, noradrenaline

There are many studies about the relation between temperature and SSNHL and suggest that the effect of temperature changes on SSNHL is similar to the mechanism by which temperature changes affect cerebrovascular disease (Lee et al. 2019b; Zhang et al. 2021b). The blood supply of the cochlea is supplied by the labyrinthine artery, which lacks collateral circulation. When endothelial injury, hypercoagulability, or blood stasis occurs, the cochlear microcirculation may be damaged, resulting in edema, ischemia, and hypoxia of the inner ear tissue, and thus hearing damage (Capaccio et al. 2007). Large temperature difference can activate the function of autonomic nerve, enhance the excitability of sympathetic nerve of inner ear, and increase sympathetic excitability of the inner ear. At the same time, it can cause vasospasm, increase platelet count, increase blood viscosity in the inner ear, and even lead to vascular embolism or thrombosis, resulting in microcirculatory disorders in the inner ear and causing SSNHL under the influence of body fluid (Lavados et al. 2018). Zhang et al. have found that the characteristics of temperature changes at onset in patients with full-frequency descending SSNHL were similar to those at onset of ischemic stroke, which further supported the pathogenesis discussed earlier: temperature may affect the inner ear microcirculation (Zhang et al. 2019a). Additionally, the temperature variation may function as a catalyst for the disruption of microcirculation in the inner ear. SSNHL was more easily induced by high DTR to low DTR. The sympathetic nervous system and the renin-angiotensin system are triggered when the body’s ability to adjust to a temperature shift is exceeded (Du et al. 2021). This increased secretion of sweat causes the increase of blood viscosity and slow blood stasis, which affects the inner ear microcirculation leading to the pathogenesis (Fig. 9).

Our study also supports the findings of previous studies that high temperatures increase the incidence of SSNHL. Besides, we also found that low DTR may reduce the onset of SSNHL in men. Further research into this phenomenon is required to conclude the potential relationship between DTR and SSNHL.

Up to now, there are few studies on the humidity and SSNHL. In our study, RH affected practically all groups. The audiogram configuration of SSNHL can be categorized into five distinct patterns: ascending, descending, flat, profound, and others (Mattox and Simmons 1977). According to a prior study, when RH levels were high, individuals were most likely to be ascending patterns. Our results also support this opinion. However, different patterns were proved to be associated with various pathogenetic mechanisms (Kuhn et al. 2011). According to a prior study, individuals with ascending patterns had RH levels that were noticeably higher on the day of commencement (Zhang et al. 2021b). Therefore, we speculate that the large effect of RH may be related to end lymphatic hydrops, although further confirmation is needed.

### Air pollution

**Fig. 10:**
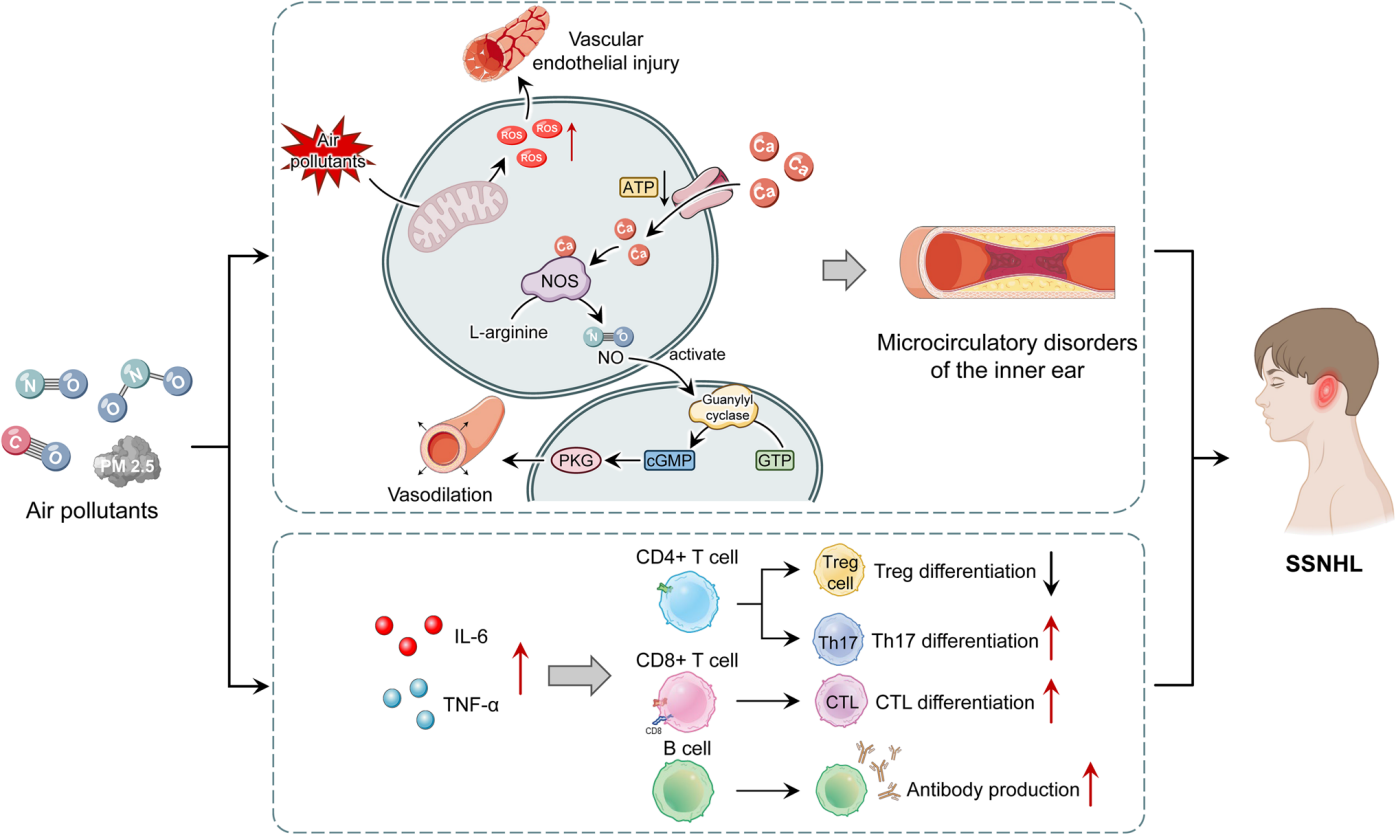
Potential mechanisms for linkage of air pollution and SSNHL. Air pollutants damage inner ear epithelial cells by inducing ROS and inflammatory responses, resulting in inner ear microcirculation disorders. ROS: when the concentration of air pollutants is high, ROS will be induced to increase. ROS depresses levels of NO, induces monocyte invasion, elevates lipid peroxidation, promotes phenotype switching of VSMCs, induces EC dysfunction, precipitates inflammation, and alters vascular responses and vasotone, which can cause vascular endothelial damage on the one hand, and on the other hand, it can promote Ca^2+^ inward flow, consume ATP, inhibit the guanylate cycle, reduce cGMP production, and cause vascular smooth muscle spasm, resulting in microcirculation disorders in the inner ear. Inflammatory immune response: Air pollutants can cause elevated levels of IL-6 and TNF and promote the activation and differentiation of CD4T cells, CD8T cells, and B cells, thereby triggering inflammatory responses. SSNHL, sudden sensorineural hearing loss; ROS, reactive oxygen species; ATP, adenosine triphosphate; cGMP, cyclic guanosine monophosphate; O_2_, oxygen; NO, nitrogen; GTP, guanosine triphosphate; PKG, protein kinase G; IL-6, interleukin 6; TNF, tumor necrosis factor; NF-κB, nuclear factor kappa-B; SIP, steroid receptor coactivator, Th17, T helper cell 17; CTL, cytotoxic T lymphocyte; CD4 + , cluster of differentiation 4 plus; CD8 + cluster of differentiation 8 plus

Industrial production increases the health and economic burden caused by air pollution (Bai et al. 2018). Air pollutants include many kinds: NO_2_, NO, CO, PM2.5, etc. Several studies suggest that air pollution could induce SSNHL by many ways including toxic effects, oxidative stress, and inflammatory pathways (Choi et al. 2019; Lee et al. 2019a; Tsai et al. 2021; Cheng et al. 2022; Tang et al. 2022). The possible specific mechanisms of this may be as follows: oxidative stress is dysregulated after inhalation of air pollutants, resulting in increased reactive oxygen species (ROS), which destroys endothelial cells, affects inner ear microcirculation, and induces SSNHL (Lehner et al. 2011); when exposed to air pollution, inflammatory cytokine expression is increased, causing an inflammatory response, thereby increasing susceptibility to infection (Hesterberg et al. 2009) (Fig. 10); in addition, air pollutants are associated with increased cardiovascular morbidity (Dehbi et al. 2017). Combined with the above association of SSNHL with cardiovascular disease, it can also be speculated that air pollution may have an impact on SSNHL. And air pollutants may also interact with each other, for example, NO_2_ can affect the concentration of NO in the cochlea, and NO, as a signaling molecule between the cochlear space and blood vessels, can lead to changes in cochlear neurotransmission and neuroregulation, which leads to hearing impairment (Heinrich and Helling 2012).

Based on the results of previous studies, we analyzed the correlation between air pollutants and MF. The results showed that the T-mean and AP were negatively associated with NO_2_, PM_2.5_, SO_2_, PM_10_, and CO and positively associated with O_3_. The DTR was positively associated with NO_2_, PM_2.5_, SO_2_, O_3_, PM_10_, and CO. However, RH was negatively associated with NO_2_, PM_2.5_, SO_2_, PM_10_, CO, and O_3_.

### Limitation and advantage

It is important to note that the present study has some limitations. First, the lack of specific information on the participants’ time spent outside, which can indicate their true degree of exposure to meteorological factors, may result in exposure measurement inaccuracies. Second, since there is a dearth of detailed information, certain possible confounding variables, such as socioeconomic position and family history, could not be adjusted. Finally, since the statistics used in this study were solely from one single city, care should be used when extrapolating the findings of our research to other areas with different climates. More epidemiological studies founded on larger-scale regions and different populations, together with mechanism findings, are urgently required to understand the specific processes responsible for the connections between meteorological variables and SSNHL onset.

Despite these limitations, our study has several advantages. Currently, there are few studies reporting the correlation between meteorological factors and SSNHL. This may be because: (1) SSNHL may appear as a standalone issue, as a systemic disease’s presenting symptom, or during an established diagnosis, which makes it difficult to diagnose, and there are few related reports. As a common disease among the elderly, hearing loss has become a serious public health problem as aging intensifies (Jiang et al. 2023). To the best of our knowledge, this is the first study to comprehensively measure and assess the effect of meteorological variables such as MT, DTR, AP, and RH on SSNHL-related admissions in China using a time-series approach. Additionally, the subgroup analysis was further conducted based on sex, age, and status of admission to identify possible vulnerable individuals and obtain a much more accurate estimation of the influence of MF on hospital admission for SSNHL. Additional epidemiological data confirming the effects of meteorological variables on SSNHL could be discovered in our current investigation. Our current investigation discussed the effects of meteorological variables, such as AP, DTR, RH, and T-mean on SSNHL based on additional epidemiological data. Finally, we collated the previous relevant literature to deeply investigate the possible effects and mechanisms of meteorological factors on SSNHL from several aspects: wind speed, air pressure, temperature, humidity, and air pollutants. Based on the results of this study, it is advisable to maintain a suitable living environment temperature and avoid extreme temperature fluctuations and high humidity. During periods of high air pollution, it is recommended to stay indoors and refrain from outdoor exercise. Individuals with underlying conditions like high blood pressure should focus on a balanced diet, and regular exercise and monitor environmental factors such as temperature, humidity, air quality, and air pressure, taking necessary precautions when needed.

## Conclusion

This is the first study to comprehensively measure and assess the effect of meteorological variables such as MT, DTR, AP, and RH on SSNHL-related admissions in China using a time-series approach. Our study concludes that exposure to high DTR and RH values, low T-mean values, and all AP grades might increase hospital admissions for SSNHL in areas with humid subtropical monsoon climates, particularly for the first admission. Interestingly, whereas women are vulnerable to all grades of AP exposure, men appear to be more sensitive to low DTR exposure, although older individuals tend to be more sensitive to high RH exposure. Therefore, it is advisable to avoid extreme temperature fluctuations and high humidity. During periods of high air pollution, it is recommended to stay indoors and refrain from outdoor exercise. Individuals with underlying conditions like high blood pressure should focus on a balanced diet, regular exercise, and monitor environmental factors such as temperature, humidity, air quality, and air pressure, taking necessary precautions when needed.

### Supplementary Information

Below is the link to the electronic supplementary material.

Supplementary file1 (DOCX 871 KB)

## Data Availability

The datasets used and/or analyzed during the current study are available from the corresponding author upon reasonable request.
