# Correction to: Association of sudden sensorineural hearing loss with meteorological factors: a time series study in Hefei, China, and a literature review

**DOI:** 10.1007/s11356-024-34299-2

**Published:** 2024-07-08

**Authors:** Xiao‑Bo Li, Yan‑Xun Han, Zi‑Yue Fu, Yu‑Chen Zhang, Min Fan, Shu‑Jia Sang, Xi‑Xi Chen, Bing‑Yu Liang, Yu‑Chen Liu, Peng‑Cheng Lu, Hua‑Wei Li, Hai‑Feng Pan, Jian‑Ming Yang

**Affiliations:** 1 Department of Otolaryngology, Head and Neck, Surgery, Second Affiliated Hospital of Anhui Medical, University, No. 678 Furong Road, Hefei, Anhui 230601 People’s Republic of China; 2 Department of Otolaryngology, Head and Neck Surgery, The First Affiliated Hospital of Anhui Medical University, 218 Jixi Road, Hefei, Anhui China; 3 Department of Clinical Medicine, Anhui Medical University, 81 Meishan Road, Hefei, Anhui China; 4 Institute and Otorhinolaryngology, Eye & ENT Hospital, State Key Laboratory of Medical Neurobiology, NHC Key Laboratory of Hearing Medicine Research, Fudan University, Shanghai, 200032 People’s Republic of China; 5 Department of Epidemiology and Biostatistics, School of Public Health, Anhui Medical University, 81 Meishan Road, Hefei, Anhui China

**Correction to: Environmental Science and Pollution Research (2024) 31:42970–429900**

10.1007/s11356-024-33943-1

The equal Author contribution statement and Acknowledgments are missing in the published proof.

Xiao-Bo Li, Yan-Xun Han and Zi-Yue Fu have contributed equally to this work and share the first authorship.

**Acknowledgements** We thank Bullet Edits Limited for the linguistic editing and proofreading of the manuscript.

The Original article has been corrected.

