# Supplementary material for: Association of sudden sensorineural hearing loss with meteorological factors: a time series study in Hefei, China, and a literature review
Source: Environ Sci Pollut Res Int. 2024 Jun 17;31(30):42970–90. doi: 10.1007/s11356-024-33943-1 (PMC11222232; doi:10.1007/s11356-024-33943-1)
Supplement: Supplementary file 1 — Supplementary file1 (DOCX 871 KB) [file 11356_2024_33943_MOESM1_ESM.docx]

**Supplement table (1):** Database and search strategy of this study.

| Data bases | Search strategy |
| --- | --- |
| PubMed | 1. Air Pollution[TIAB] |
|  | 2. Air Pollutions[TIAB] OR Pollution, Air[TIAB] OR Air Quality[TIAB] |
|  | 3. 1 OR 2 |
|  | 4. Meteorological Concepts[TIAB] |
|  | 5. Concept, Meteorological[TIAB] OR Concepts, Meteorological[TIAB] OR Meteorological Concept[TIAB] OR Meteorological Factors[TIAB] OR Factor, Meteorological [TIAB] OR Factors, Meteorological [TIAB] OR Meteorological Factor [TIAB] OR Meteorologic Factors [TIAB] OR Factor, Meteorologic [TIAB] OR Factors, Meteorologic [TIAB] OR Meteorologic Factor [TIAB] |
|  | 6. Relative Humidity[TIAB] OR RH[TIAB] OR Atmospheric Pressure[TIAB] OR AP[TIAB] OR Temperature[TIAB] |
|  | 7. 4 OR 5 OR 6 |
|  | 8. 3 OR 7 |
|  | 9. Hearing Loss, Sudden[TIAB] |
|  | 10. Sudden Hearing Loss[TIAB] OR Deafness, Sudden [TIAB] OR Sudden Deafness[TIAB] |
|  | 11. Sudden Sensorineural Hearing Loss OR SSNHL OR Idiopathic Sudden Deafness OR Idiopathic Sudden Sensorineural Hearing Loss OR ISSNHL |
|  | 12. 9 OR 10 OR 11 |
|  | 13. 8 AND 12 |
| The Cochrane Library | #1 (Air Pollution) ab,ti,kw |
|  | #2 (Air Pollutions)ab,ti,kw OR( Pollution, Air) ab,ti,kw OR (Air Quality)ab,ti,kw |
|  | #3 #1 OR #2 |
|  | #4 (Meteorological Concepts)ab,ti,kw |
|  | #5 (Concept, Meteorological)ab,ti,kw OR (Concepts, Meteorological)ab,ti,kw OR (Meteorological Concept)ab,ti,kw OR (Meteorological Factors)ab,ti,kw OR (Factor, Meteorological)ab,ti,kw OR (Factors, Meteorological) ab,ti,kw OR (Meteorological Factor)ab,ti,kw OR (Meteorologic Factors) ab,ti,kw OR (Factor, Meteorologic) ab,ti,kw OR (Factors, Meteorologic) ab,ti,kw OR (Meteorologic Factor) ab,ti,kw OR (Relative Humidity)ab,ti,kw OR (RH)ab,ti,kw OR (Atmospheric Pressure)ab,ti,kw OR (AP)ab,ti,kw OR (Temperature)ab,ti,kw |
|  | #6 #4 OR #5 |
|  | #7 #3 OR #6 |
|  | #8. (Deafness, Sudden):ab,ti,kw OR (Hearing Loss, Sudden):ab,ti,kw OR (Sudden Hearing Loss):ab,ti,kw OR (Sudden Deafness):ab,ti,kw OR (Sudden Sensorineural Hearing Loss):ab,ti,kw OR (SSNHL):ab,ti,kw OR (Idiopathic Sudden Deafness):ab,ti,kw OR (Idiopathic Sudden Sensorineural Hearing Loss):ab,ti,kw OR (ISSNHL):ab,ti,kw |
|  | #9.#7 AND #8 |
| Web of Science | 1. TS=( Air Pollution OR Air Pollutions OR Pollution, Air OR Air Quality) |
|  | 2. TS=(Concept, Meteorological OR Concepts, Meteorological OR Meteorological Concept OR Meteorological Factors OR Factor, Meteorological OR Factors, Meteorological OR Meteorological Factor OR Meteorologic Factors OR Factor, Meteorologic OR Factors, Meteorologic OR Meteorologic Factor OR Meteorological Concepts OR Relative Humidity OR RH OR Atmospheric Pressure OR AP OR Temperature) |
|  | 3. 1 OR 2 |
|  | 4. TS=(Hearing Loss, Sudden OR Sudden Hearing Loss OR Deafness, Sudden OR Sudden Deafness OR Sudden Sensorineural Hearing Loss OR SSNHL OR Idiopathic Sudden Deafness OR Idiopathic Sudden Sensorineural Hearing Loss OR ISSNHL) |
|  | 5. 3 AND 4 |
| Embase | #1 ‘Air Pollution’:ab,ti |
|  | #2 ‘Aerial Pollution’:ab,ti OR ‘Aerogenic Pollution’:ab,ti OR ‘Air Contamination’:ab,ti OR ‘Air Pollutioning’:ab,ti OR ‘Air-borne Pollution’:ab,ti OR ‘Airborne Pollution’:ab,ti OR ‘Atmosphere Pollution’:ab,ti OR ‘Atmospheric Pollution’:ab,ti OR ‘Polluted Air’:ab,ti OR ‘Polluted Atmosphere’:ab,ti OR ‘Pollution, Air’:ab,ti |
|  | #3 #1 OR #2 |
|  | #4 ‘Meteorological Phenomena’ :ab,ti |
|  | #5 ‘Meteorological Concepts’:ab,ti OR ‘Meteorological Factors’:ab,ti OR ‘Relative Humidity’:ab,ti OR ‘RH’:ab,ti OR ‘Atmospheric Pressure’:ab,ti OR ‘AP’:ab,ti OR ‘Temperature’:ab,ti |
|  | #6 #4 OR #5 |
|  | #7 #3 OR #6 |
|  | #8 'Deafness, Sudden':ab,ti OR 'Hearing Loss, Sudden':ab,ti OR 'sudden Hearing Loss':ab,ti OR 'Sudden Deafness':ab,ti OR 'Sudden Sensorineural Hearing Loss':ab,ti OR 'SSNHL':ab,ti OR 'Idiopathic Sudden Deafness':ab,ti OR 'Idiopathic Sudden Sensorineural Hearing Loss':ab,ti OR 'ISSNHL':ab,ti  #9 #7 AND #8 |

**Supplement table (2):** Abbreviation Table.

| **Abbreviation** | **Definition** |
| --- | --- |
| SSNHL | sudden sensorineural hearing loss |
| T-mean | temperature mean |
| DTR | diurnal temperature range |
| AP | atmospheric pressure |
| RH | relative humidity |
| RR | relative risk |
| SD | standard deviation |
| RMB | renminbi |
| WS | wind speed |
| PM_2.5_ | particulate matter ≤ 2.5 μm in aerodynamic diameter |
| PM_10_ | particulate matter ≤ 10 μm in aerodynamic diameter |
| SO_2_ | sulfur dioxide |
| NO_2_ | nitrogen dioxide |
| CO | carbon monoxide |
| O_3_ | ozone |

Abbreviations: SD: standard deviation; Tmean: temperature mean; DTR: diurnal temperature range; AP: atmospheric pressure; WS: wind speed; PM2.5: particulate matter ≤ 2.5 μm in aerodynamic diameter; PM10: particulate matter ≤ 10 μm in aerodynamic diameter; SO2: sulfur dioxide; NO2: nitrogen dioxide; CO: carbon monoxide; O3: ozone

**Supplementary Fig (1):** Time series of SSNHL, T-mean, diurnal temperature range, relative humidity, and atmospheric pressure in Hefei, China, from 2014 to 2021.


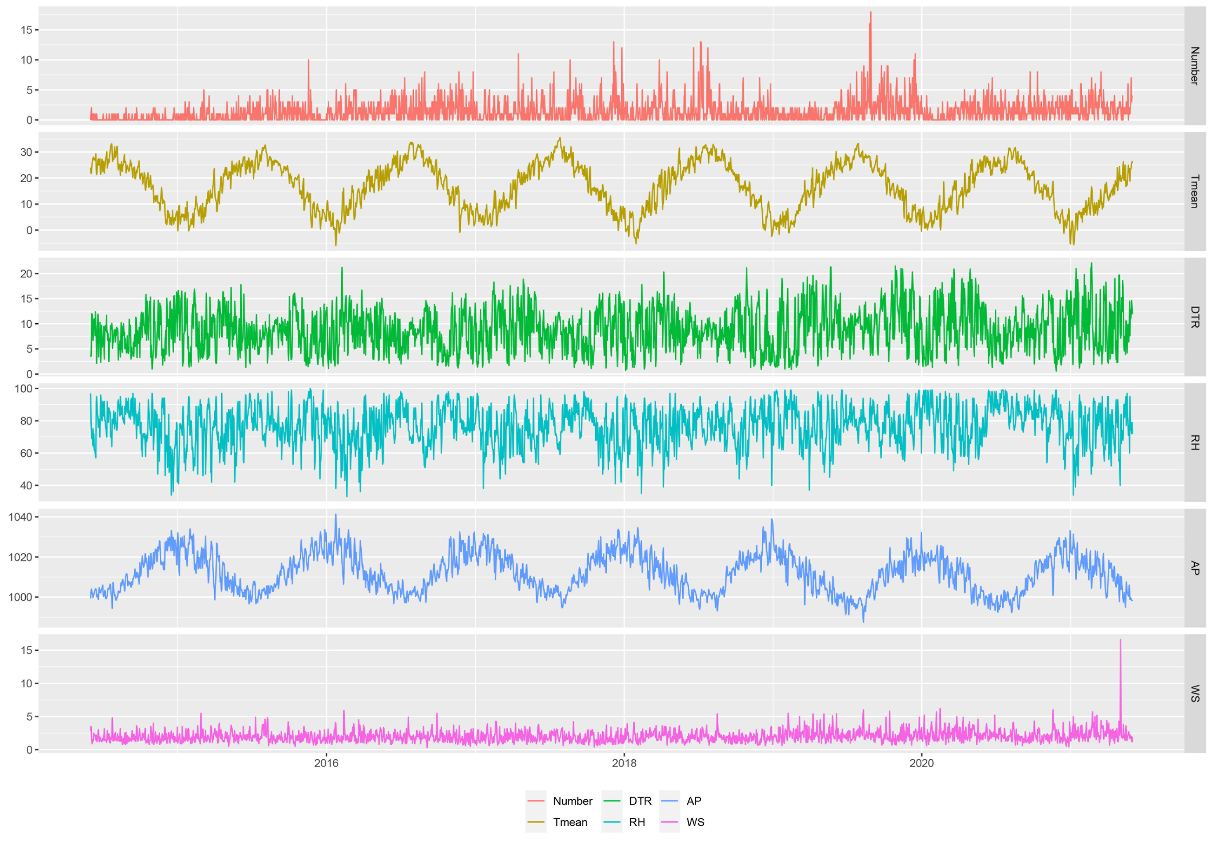


**Abbreviations: SSNHL,** sudden sensorineural hearing loss; T-mean, temperature mean.
